# Metabolomics profiling reveals differences in proliferation between tumorigenic and non-tumorigenic Madin-Darby canine kidney (MDCK) cells

**DOI:** 10.7717/peerj.16077

**Published:** 2023-09-20

**Authors:** Na Sun, Yuchuan Zhang, Jian Dong, Geng Liu, Zhenbin Liu, Jiamin Wang, Zilin Qiao, Jiayou Zhang, Kai Duan, Xuanxuan Nian, Zhongren Ma, Xiaoming Yang

**Affiliations:** 1 Gansu Technology Innovation Center of Animal Cell, Biomedical Research Center, Northwest Minzu University, Lanzhou, China; 2 Engineering Research Center of Key Technology and Industrialization of Cell-based Vaccine, Ministry of Education, Lanzhou, China; 3 Gansu Provincial Bioengineering Materials Engineering Research Center, Lanzhou, China; 4 Wuhan Institute of Biological Products Co., Ltd., Wuhan, China; 5 National Engineering Technology Research Center for Combined Vaccines, Wuhan, China; 6 Key Laboratory of Biotechnology and Bioengineering of National Ethnic Affairs Commission, Biomedical Research Center, Northwest Minzu University, Lanzhou, China; 7 China National Biotech Group Company Limited, Beijing, China

**Keywords:** MDCK cells, Cell proliferation, Untargeted metabolomics, Targeted metabolomics, Tryptophan metabolism, S-adenosylmethionine, Choline metabolism, MAT, IDO

## Abstract

**Background:**

Madin-Darby canine kidney (MDCK) cells are a cellular matrix in the production of influenza vaccines. The proliferation rate of MDCK cells is one of the critical factors that determine the vaccine production cycle. It is yet to be determined if there is a correlation between cell proliferation and alterations in metabolic levels. This study aimed to explore the metabolic differences between MDCK cells with varying proliferative capabilities through the use of both untargeted and targeted metabolomics.

**Methods:**

To investigate the metabolic discrepancies between adherent cell groups (MDCK-M60 and MDCK-CL23) and suspension cell groups (MDCK-XF04 and MDCK-XF06), untargeted and targeted metabolomics were used. Utilizing RT-qPCR analysis, the mRNA expressions of key metabolites enzymes were identified.

**Results:**

An untargeted metabolomics study demonstrated the presence of 81 metabolites between MDCK-M60 and MDCK-CL23 cells, which were mainly affected by six pathways. An analysis of MDCK-XF04 and MDCK-XF06 cells revealed a total of 113 potential metabolites, the majority of which were impacted by ten pathways. Targeted metabolomics revealed a decrease in the levels of choline, tryptophan, and tyrosine in MDCK-CL23 cells, which was in accordance with the results of untargeted metabolomics. Additionally, MDCK-XF06 cells experienced a decrease in 5’-methylthioadenosine and tryptophan, while S-adenosylhomocysteine, kynurenine, 11Z-eicosenoic acid, 3-phosphoglycerate, glucose 6-phosphate, and phosphoenolpyruvic acid concentrations were increased. The mRNA levels of MAT1A, MAT2B, IDO1, and IDO2 in the two cell groups were all increased, suggesting that S-adenosylmethionine and tryptophan may have a significant role in cell metabolism.

**Conclusions:**

This research examines the effect of metabolite fluctuations on cell proliferation, thus offering a potential way to improve the rate of MDCK cell growth.

## Introduction

Influenza is a highly transmittable respiratory disorder caused by the influenza virus, with an estimated global death toll of between 290,000 and 650,000 annually (Trombetta et al., 2022). Vaccination is the most effective and cost-efficient way to prevent the transmission of the virus. In addition to the traditional chicken embryo culture method for vaccine production, cell culture techniques are increasingly being employed, such as Vero cells for the production of COVID-19 vaccines (Zhang et al., 2022), purified chick embryo cell-culture for rabies vaccines (PCECV; Preiss et al., 2018), and PER.C6 cells for the production of poliovirus vaccines (Sanders et al., 2013).

Madin and Darby first isolated and established the MDCK cell line from a healthy cocker spaniel’s kidney (Ye et al., 2022). This cell line has been found to be sensitive to multiple strains of the influenza virus, making it the primary host cell line for influenza vaccine production (Abdoli et al., 2016; Donis et al., 2014; Liu et al., 2010). However, Omeir et al. (2011) discovered that all batches of MDCK cells could cause tumors in nude mice, posing a potential safety hazard for vaccine production. To address this issue, an adherent non-tumorigenic MDCK cell line (MDCK-CL23) was cloned from a heterologous cell population, and then domesticated into a suspension MDCK-XF06 cell. Unfortunately, studies have shown that the proliferation rate of these cells is slower than that of their parent tumorigenic MDCK cells, which could affect vaccine production efficiency (Ma et al., 2020). It is therefore imperative to understand the molecular basis of MDCK cell proliferation.

Metabolomics has become an increasingly important tool in systems biology capable of reflecting cell phenotypes (Zhang et al., 2013). Metabolomics provides insight into the link between metabolites and physiological changes, and has been used to elucidate cell metabolism, including cell differentiation and proliferation (Garcia-Manteiga et al., 2011; Sun et al., 2022; Zheng et al., 2021). For example, Sun et al. (2018) performed a metabolomics investigation to examine the inhibitory role of magnoline on the proliferation of the prostate cancer cell line 22RV1, finding that magnoline inhibited the growth of the cells by affecting 12 metabolic biomarkers. Lu et al. (2022) used metabolomics to elucidate the function of Atrazine (ATZ) in the proliferation of MCF-7 cells, identifying 34 metabolites with significant changes that were related to the proliferation of the cells. Sunaga, Kofuji & Nishina (2021) employed metabolomics to study the mechanism of cell competition dependent on the Yes-associated protein (YAP), demonstrating that YAP is essential to cell competition through choline metabolism activation. These studies show that metabolomics is an effective approach to uncovering the mechanisms of cell proliferation.

This study employed untargeted metabolomics based on ultraperformance liquid chromatography-tandem mass spectrometry (LC-MS/MS) and targeted metabolomics based on ultraperformance liquid chromatography coupled to quadrupole time-of-flight mass spectrometry (UHPLC-QTRAP-MS) to analyze metabolic discrepancies between high proliferation and low proliferation MDCK cells, with the aim of screening small intracellular molecular metabolites. RT-qPCR was used to identify the mRNA level of related metabolic enzymes to further verify the metabolomics results. Our findings add new insights for uncovering the possible mechanism behind a low proliferation of MDCK cells and exploring potential biomarkers for cell proliferation, toward the goal of improving cell density based on the vaccine being produced.

## Materials & Methods

### Cell lines

The MDCK-M60 cell line was established as the primary cell bank derived from the MDCK cell line (obtained from ATCC, CCL-34). Additionally, the adherent MDCK-CL23 cell line was generated and stored in-house, which is a MDCK-M60-derived monoclonal cell line. The cells were kept in DMEM containing 10% newborn bovine serum (NBS; Lanzhou Minhai Bio-engineering Co., Ltd, Lanzhou, China) at 37 °C and 5% CO_2_. MDCK-XF04 and MDCK-XF06, two suspension cell lines, were adapted from the adherent MDCK-M60 and MDCK-CL23 cells, respectively, and cultured in a serum-free medium (Lanzhou Bailing Biotechnology Co., Ltd, Lanzhou, China) in a shaker at 37 °C and 5% CO_2_.

### Sample collection and metabolite extraction

For cell sample preparation, adherent MDCK cells were seeded in T75 cell culture flaps, washed twice with PBS after growing more than 95% confluence, then collected and transferred to a 1.5 mL centrifuge tube. When the density of the suspended cells reached 8 million/mL, the suspension cells were counted and collected in a 15 mL centrifuge tube. The total number of cells was 1 × 10^7^. The cell samples were kept in an ultra-cold storage freezer at −80 °C. For metabolite extraction, 25 mg of the cell sample was slowly thawed at 4 °C, then added to an Eppendorf tube containing 800 µL of a cold extraction solution (methanol: acetonitrile: water = 2: 2:1, v:v:v) and 10 µL of an internal standard. Using a tissue grinder (50 Hz, 5min), the mixture was stirred and homogenized. Following a 10-minute sonication in a cold-water bath, the samples were cooled to −20 °C for 1 h and then centrifuged at 25,000 rpm at a low temperature for 15 min. The supernatant liquid (600 µL) was extracted and dried using a chilled vacuum concentrator. The solution was then redissolved with 200 µL of a reconstitution fluid composed of a 1:9 methanol-water ratio, volume-to-volume. The solution underwent a 1-minute vortexing process and was then subjected to 10 min of ultrasonic treatment. To assess the accuracy and reliability of the LC-MS analysis process, a quality control (QC) sample was created by combining the supernatant of each sample.

### Untargeted metabolomics

The BEH C18 column (1.7 µm 2.1 × 100 mm; Waters, Milford, MA, USA) was selected with a column temperature of 45 °C and a flow rate of 0.35 mL/min. The injection volume was set at 5 µL. The mobile phase for the negative ion mode consisted of 10 mM ammonium formate in 95% methanol and 10 mM ammonium formate in water. The eluent for the positive ion mode consisted of 0.1% formic acid in 100% methanol and 0.1% formic acid in water. The elution gradient was as follows: 2% B from 0 to 1 min, 2% to 98% B from 1 to 9 min, 98% B from 9 to 12 min, 98% to 2% B from 12 to 12.1 min, and 2% B from 12.1 to 15 min.

The Q Exactive HF mass spectrometer (Thermo Fisher Scientific, Waltham, MA, USA) was used to acquire the primary and secondary spectra of the samples, with a sheath gas flow rate of 40, an aux gas flow rate of 10, a spray voltage of 3.80 KV (ESI+) and 3.20 KV (ESI-), a capillary temperature of 320 °C, and an aux gas heater temperature of 350 °C.

The raw LC-MS/MS data were preprocessed using MetaboAnalys 5.0 and then submitted to Compound Discoverer 3.1 (Thermo Fisher Scientific, Waltham, MA, USA) for peak alignment, peak extraction, and other processes. A principal Component Analysis (PCA) and Partial Least Squares Discriminant Analysis (PLS-DA) were then performed on the data. Metabolite identification was matched against various databases, such as the BMDB Database (BGI MDB, BGI Metabolome Database, China), HMDB (https://www.hmdb.ca), mzCloud (https://www.mzcloud.org/), LipidMaps (https://lipidmaps.org/), KEGG (https://www.genome.jp/kegg) and ChemSpider (http://www.chemspider.com/). The Fold Change (FC) results from the univariate analysis and Student’s *t*-test and the Variable Importance (VIP) values of the first two principal components of the PLS-DA model were used to screen the differential metabolites. Metabolites with VIP values greater than 1 were deemed to have a significant influence on group classification.

### Targeted metabolomics

A targeted metabolomics analysis was conducted on MDCK cells to determine the difference in cell metabolites between high and low proliferation rates. A UHPLC system (Waters, USA) coupled with a QTrap 6500+ MS/MS system (AB Sciex, USA) was used for the analysis. The temperature of the HILIC column was set to 35 °C, and the flow rate and injection volume were 0.3 mL/min and 2 µL, respectively. The eluent was a combination of solutions A (90% H_2_O + 2 mM ammonium formate + 10% acetonitrile) and B (0.4% formic acid in methanol). The gradient elution procedure was employed, with 85% B at 0–1.0 min; 85–80% B at 1.0–3.0 min; 80% B at 3.0–4.0 min; 80–70% B at 4.0–6.0 min; 70–50% B at 6.0–10.0 min; 50% B at 10–15.5 min; 50–85% B at 15.5–15.6 min; and 85% B at 15.6–23 min. The BEH C18 column temperature was maintained at 40 °C, with a flow rate of 0.4 mL/min and an injection volume of 2 µL. Solutions A (0.2% NH_3_⋅ H_2_O in water and 5 mM ammonium acetate) and B (99.5% acetonitrile + 0.5% NH_3_⋅ H_2_O) constituted the mobile phase, with a gradient of 0 min, 5% B; 5 min, 60% B; 11 min, 100% B; 13 min, 100% B; and 13.1 min, 5% B. The samples were maintained at a temperature of 4 °C during the entire analysis. The AB SCIEX QTRAP 6500+ was used in two modes, positive and negative switch, with a source temperature of 580 °C. The ion source gas settings were: GS1 at 45, GS2 at 60, CUR at 35, and IS either +4500 V (ESI+) or −4500 V (ESI-). Mass spectrum data was measured using the MRM method.

MRM data was processed using the MultiQuant software to extract the peak area and interior of each substance. The standard curve was then used to calculate the ratio between the standard peak area and content. OPLS-DA and PCA were performed using the SIMCA-P software (version 14.1, Umetrics, Umea, Sweden). Differential metabolites were determined according to *p* < 0.05 and FC > 1, and volcano plots and a KEGG enrichment analysis were conducted.

### Verification of key enzymes by RT-qPCR

To verify the related gene expression of key enzymes based on targeted metabolomics pathways, four gene expressions of several kinases, including methionine adenosyltransferase (MAT1A and MAT2B) and indoleamine 2,3-dioxygenase (IDO1 and IDO2), were assessed.

Total RNA was extracted from the cell sample using Trizol reagent (AG, Hunan, China), according to the manufacturer’s directions. Following total RNA extraction, the Evo M-MLV RT Mix Kit with gDNA Clean for qPCR kit (AG, Hunan, China) was used to reverse transcribe the RNA into its complementary template DNA. Then, RT-qPCR was conducted using 2X Universal SYBR Green Fast qPCR Mix (ABclonal, Wuhan, China). The results were analyzed on a Bio-Rad CFX96 instrument (Bio-Rad). The primer sequences are available in the supporting material, and all primers used are listed in Supplemental Table S1.

### Cell proliferation

To assess the effects of choline chloride on cell proliferation, cells were plated in triplicate at a density of 5  × 10^3^ in a 96-well plate. Then, concentrations of choline chloride (Sigma Aldrich, St. Louis MO, USA) with different gradients—10, 20, 40, 80, 100, 200, and 300 µM —were added. After 24 h and 48 h of drug exposure, cells were estimated by colorimetric assay (Meilunbio, Dalian, China). The absorbance at 450 nm was measured with a spectrophotometric plate reader (Thermo Fisher Scientific, Waltham, MA, USA) and the experiment was repeated twice to ensure reproducibility. The cell viability of tryptophan (Sigma Aldrich, St. Louis MO, USA) was also performed (concentration: 50, 100, 200, 400, 600 and 800 µM).

The cells at the logarithmic growth stage were inoculated in 12-well plates with 5  × 10^3^ cells per well and three parallel wells at each time point for a total of six days. The suspension cells were inoculated at an initial density of 1  × 10^6^ cells/ml. The samples were counted every 24 h and cell growth curves were plotted to compare the specific growth rates of two different cell lines. Then, a 12-well plate with 1  × 10^4^ cells per well was set. After cell adhesion, 40 µM choline chloride was added and cultured for six days. Suspension cells were then inoculated into serum-free medium containing 40 µM choline chloride and 100 µM tryptophan at an initial density of 1  × 10^6^ cells/ml for five days, respectively. Control group cells were not treated. The cells stained with Trypan blue were placed in a Countstar (Shanghai, China) cell counter to measure cell viability and density.

### Data processing and statistical analysis

The statistical analysis was performed using the GraphPad Prism 9.0 software (GraphPad Software, Inc., San Diego, CA, USA) and the data was displayed as the mean ± standard deviation (SD). The statistical analysis was conducted using one-way analysis of variance (ANOVA) or a two-tailed Student’s *t*-test. A significance level of *p < 0.05, **p < 0.01, and ***p < 0.001 was used to define statistical significance.

## Results

### Untargeted metabolomics analysis

#### Multivariate statistical analysis

**Figure 1:**
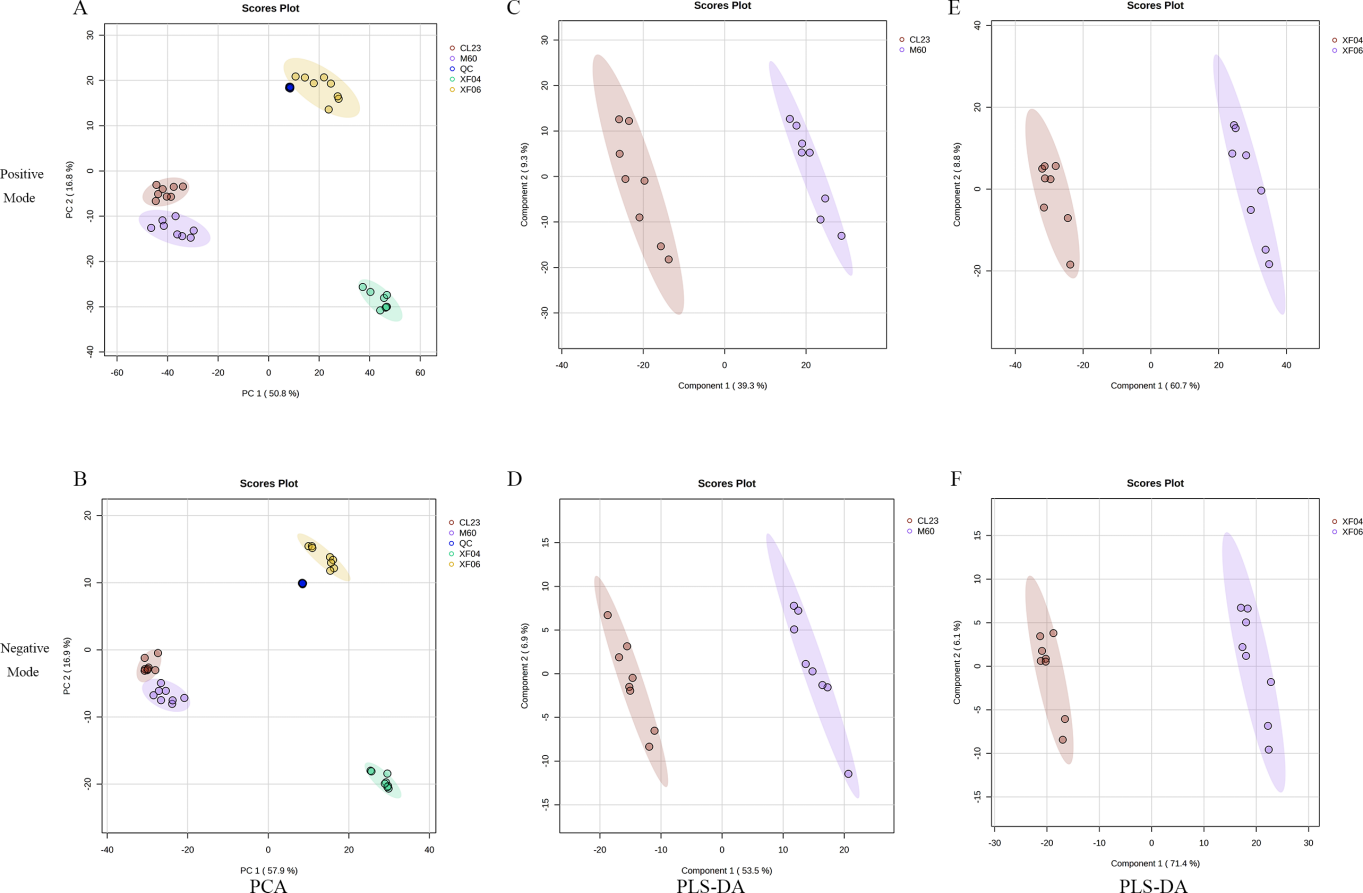
Multivariate statistical analysis of untargeted metabolomics. Scatter plots of the PCA model based on all identified metabolite features of MDCK cell samples. (A, B) PCA score plots in positive ion mode and negative ion mode among groups; (C, D) PLS-DA score plots in positive ion mode and negative ion mode (CL23 *vs* M60); (E, F) PLS-DA score plots in positive ion mode and negative ion mode (XF06 *vs* XF04).

To analyze the difference in metabolism between tumorigenic and non-tumorigenic MDCK cells, UPLC-MS/MS was used to detect four groups of cell samples. QC samples were used to measure the instrument’s stability, indicating that the data collected was reproducible and reliable. The PCA score plots of the positive and negative ion modes demonstrated a notable segregation for the four cell samples and a distinct cluster of QC samples (Figs. 1A, 1B). The PLS-DA score plot provided a satisfactory data division in the adherent (Figs. 1C, 1D) and suspension cells (Figs. 1E, 1F). The results indicated a distinct difference in the intracellular metabolites of high and low-proliferation MDCK cells.

#### Differential metabolite analysis

Using UPLC-MS/MS, differential metabolites were screened based on the criteria: FC ≥ 1.2 or FC ≤ 0.83, VIP ≥ 1, and *q*-value < 0.05. The results showed that MDCK-M60 and MDCK-CL23 cell groups had 990 and 432 metabolites that were distinct in the positive and negative ion modes, respectively. In the positive ion mode, 1487 metabolites were identified that differed between the MDCK-XF04 and MDCK-XF06 cell groups, while 552 metabolites were found to be distinct in the negative ion mode. After further analysis, 81 and 113 molecules were identified as potential biomarkers in the adherent and suspension cell groups, respectively (metabolites are provided in Tables S2 and S3).

**Figure 2:**
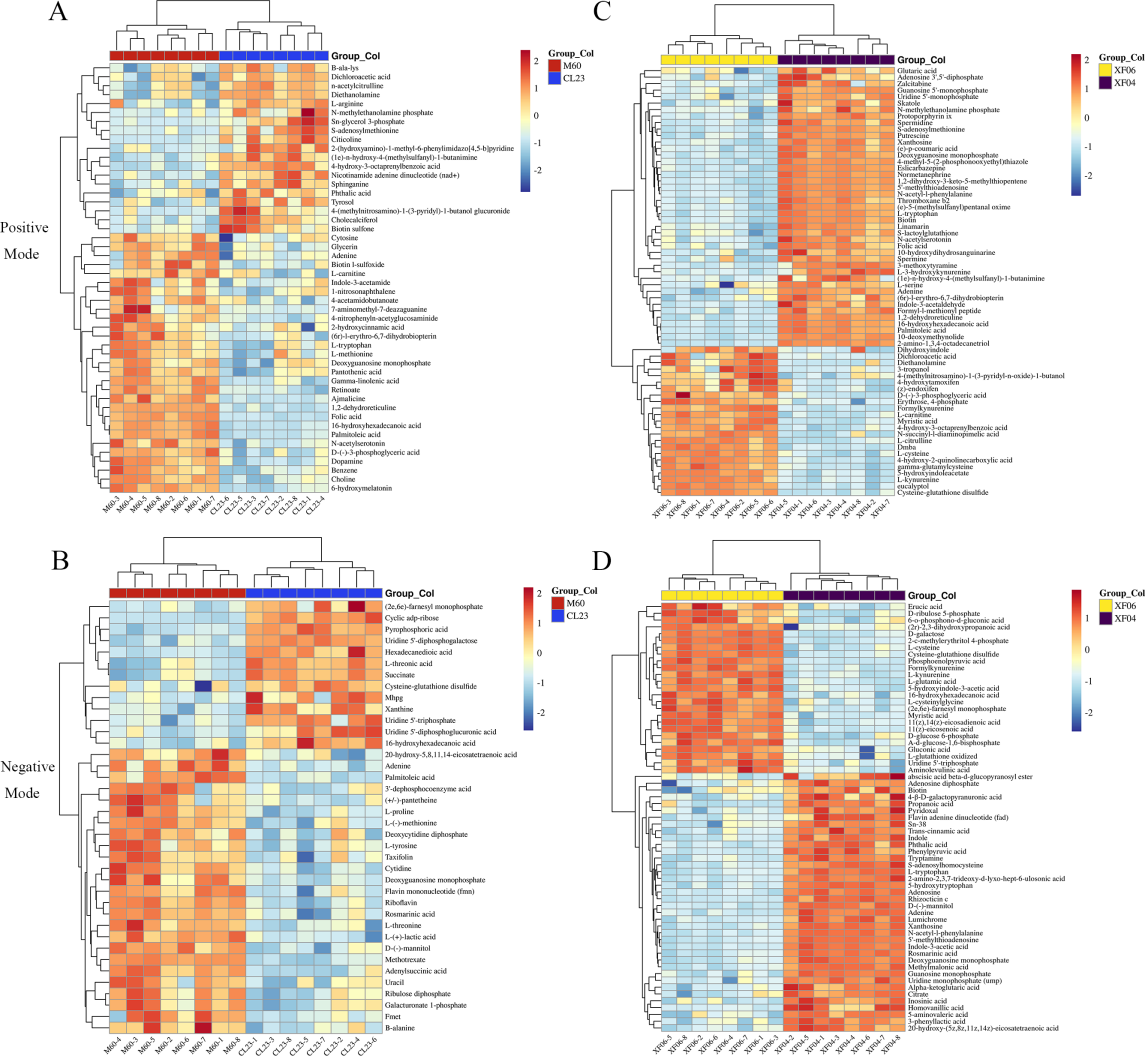
Heatmap analysis of potential metabolites. (A, B) Heatmap analysis of the relative expression data of adherent cells in positive and negative ion modes (CL23 *vs* M60). (C, D) Heatmap analysis of the relative expression data of suspension cells in positive and negative ion mode (XF06 *vs* XF04).

Further analysis revealed that 14 metabolites were upregulated more than five-fold, and 15 metabolites were downregulated to below 0.2-fold compared to the MDCK-M60 cell group. Heatmaps were then created to display the differences in the concentration of potential metabolites (Fig. 2). These metabolites included: S-adenosylmethionine (30.15-fold), citicoline (20.76-fold), nicotinamide adenine dinucleotide (8.26-fold), Sn-glycerol 3-phosphate (7.45-fold), 6-hydroxymelatonin (0.03-fold), folic acid (0.09-fold), and D-(-)-3-phosphoglyceric acid (0.14-fold). Compared to the MDCK-XF04 cell group, 33 metabolites were upregulated more than five-fold, and 34 metabolites were downregulated to below 0.1-fold. These metabolites included: L-kynurenine (33.96-fold), 5-hydroxyindoleacetate (33.47-fold), formylkynurenine (28.93-fold), N-succinyl-l-diaminopimelic acid (20.25-fold), cysteine-glutathione disulfide (19.00-fold), L-cysteine (10.32-fold), and phosphoenolpyruvic acid (87.20-fold). The metabolites with a large fold change had a greater discriminative effect between groups and could potentially be used as markers to influence cell proliferation. These differential metabolites belonged to amino acids, lipids, vitamins, and their derivatives.

#### Metabolic pathway analysis

**Figure 3:**
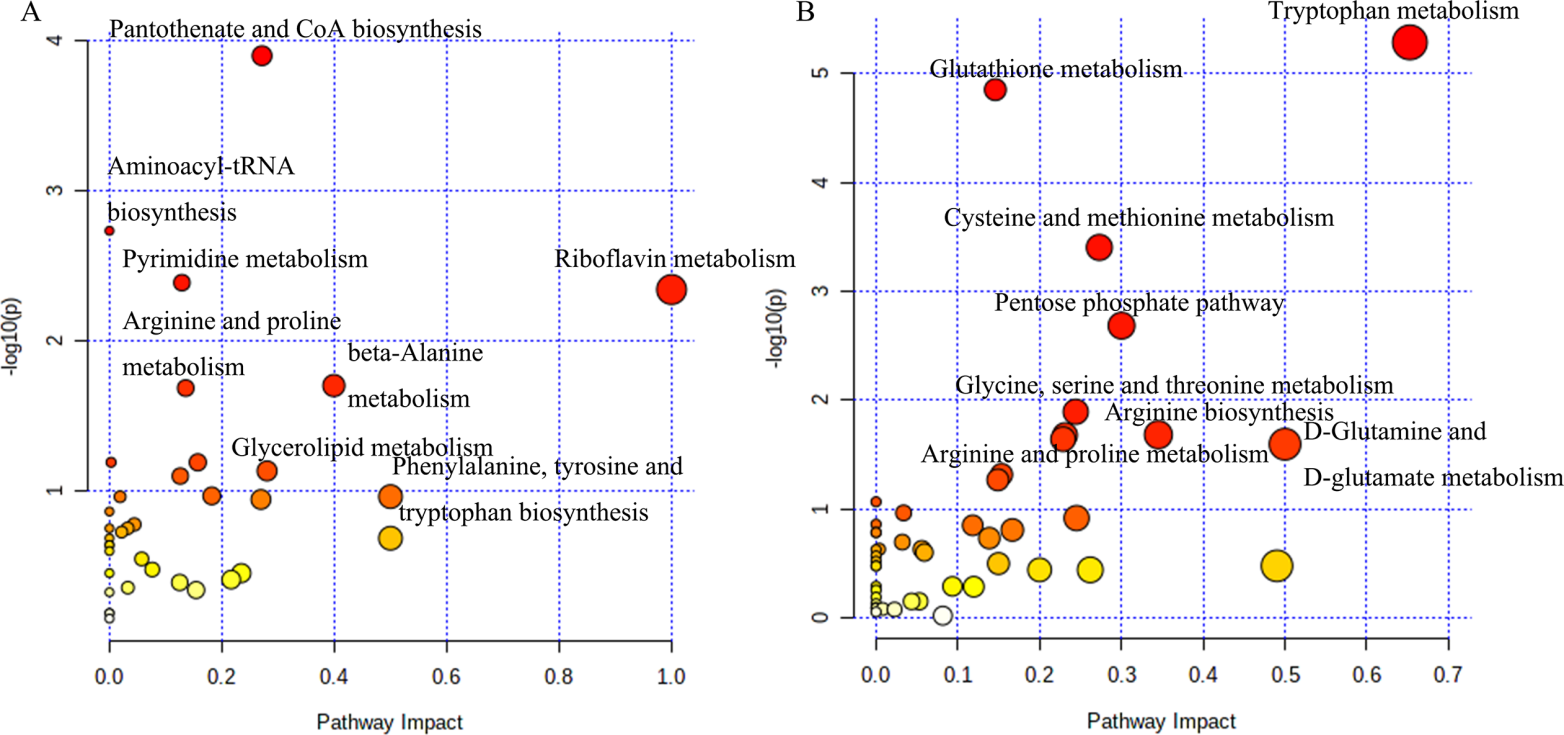
Analysis of the metabolic pathways. Differentiated metabolic pathway analysis using MetaboAnalyst 5.0. (A) CL23 *vs* M60; (B) XF06 *vs* XF04.

The analysis of potential metabolites in MetaboAnalyst 5.0 revealed significant differences in metabolic pathways between tumorigenic and non-tumorigenic MDCK cells. As shown in Fig. 3A, in the adherent cell groups, six pathways were altered: pantothenate and CoA biosynthesis, pyrimidine metabolism, aminoacyl-tRNA biosynthesis, arginine and proline metabolism, riboflavin metabolism, and β-alanine metabolism. The main metabolic pathways in the suspension cell groups (Fig. 3B) included: glutathione metabolism, tryptophan metabolism, pentose phosphate pathway, cysteine and methionine metabolism, arginine biosynthesis, glycine, serine and threonine metabolism, D-glutamine, and D-glutamate metabolism, purine metabolism, arginine, and proline metabolism, and glyoxylate and dicarboxylate metabolism.

### Targeted metabolomics analysis

**Figure 4:**
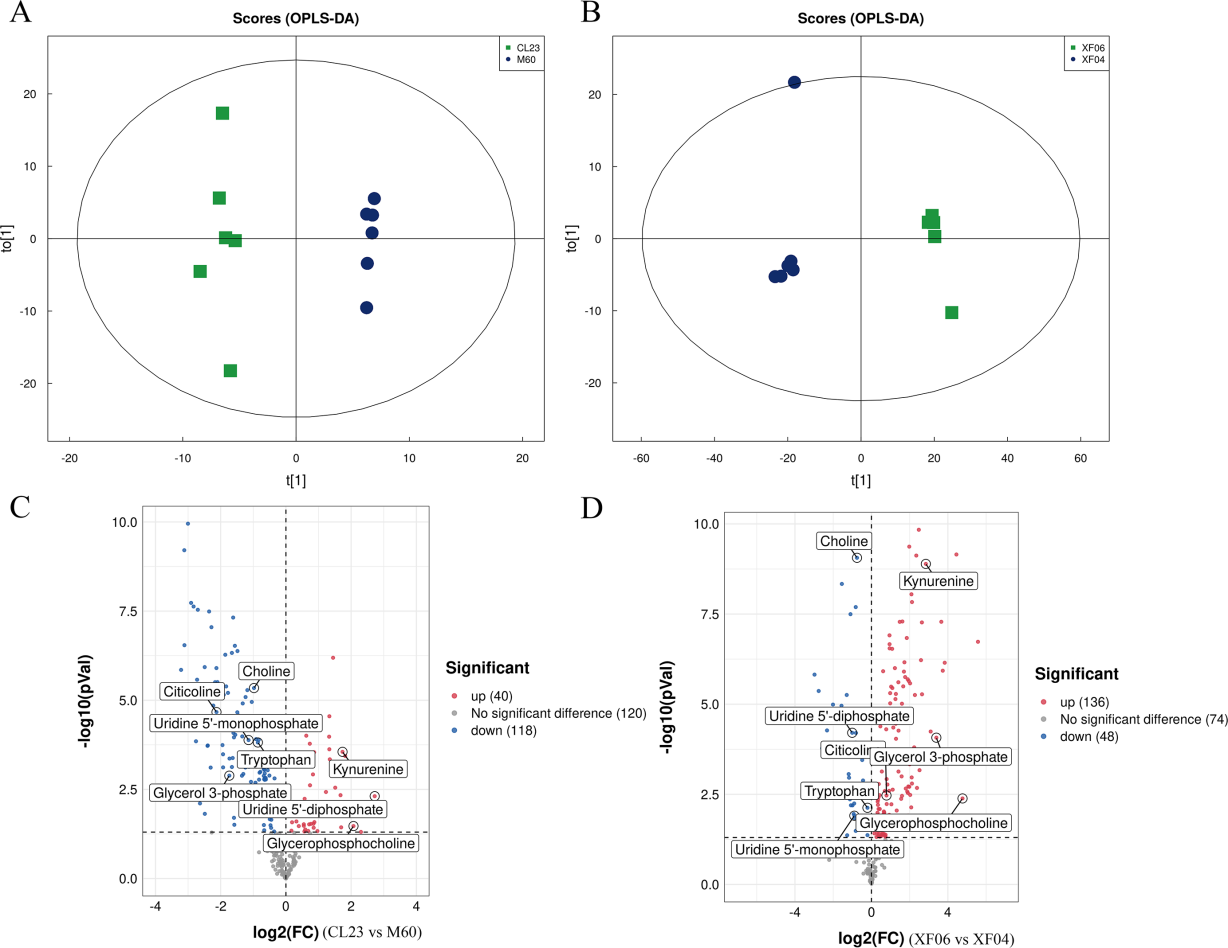
Multivariate statistical analysis of the targeted metabolomics based on UHPLC-QTRAP MS. (A, B) The OPLS-DA score plot of the adherent cell group and suspension cell groups, respectively. (C, D) Volcano plots of adherent and suspension cell groups, respectively.

A targeted metabolomics analysis was conducted using the UPLC-QTRAP-MS platform to detect the content of metabolites in the adherent and suspension cell groups. Differences between the cell groups were identified by PCA and OPLS-DA, and a permutation test was conducted to validate the models (Figs. S2A, S2B). The PCA and OPLS-DA score plots showed a clear separation between cell groups and good reproducibility and reliability of the model (Figs. 4A, 4B). A volcano plot was then mapped to show the differential metabolites between MDCK-M60 and MDCK-CL23 cells (Fig. 4C) and between the MDCK-XF04 and MDCK-XF06 cell groups (Fig. 4D). In the adherent cell groups, 40 metabolites were increased and 118 were decreased, while in the suspension cell groups, 136 metabolites were elevated and 48 were reduced.

**Figure 5:**
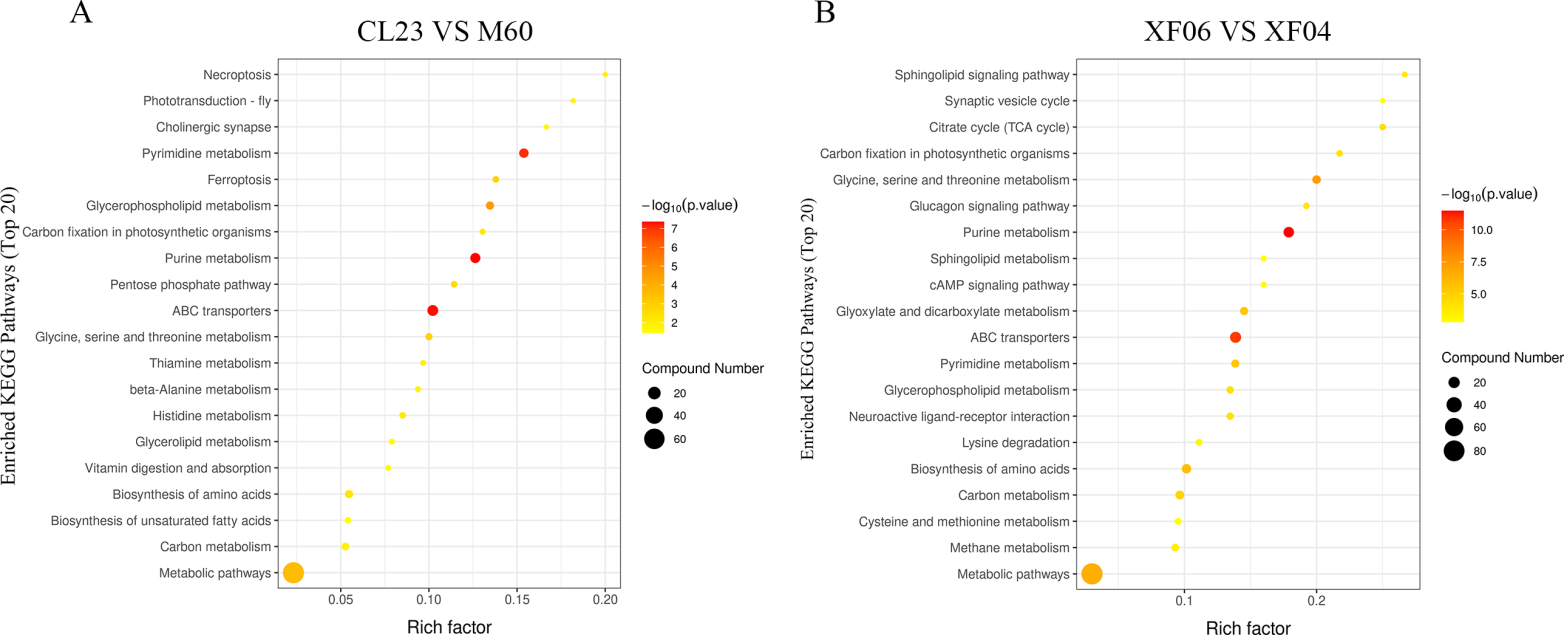
The top 20 pathways analyzed for the KEGG enrichment pathways. (A) Enrichment analysis of CL23 *vs* M60; and (B) XF06 *vs* XF04. Larger size and darker colors represent greater pathway enrichment and higher pathway impact values, respectively.

The KEGG enrichment pathway analysis revealed that the top 20 pathways for the adherent cell groups were primarily focused on pyrimidine metabolism, purine metabolism, ABC transporters, and glycerophospholipid metabolism (Fig. 5A). Similarly, in the suspension cell groups, the most prominent pathways were purine metabolism, ABC transporters, and glycine, serine and threonine metabolism (Fig. 5B).

**Figure 6:**
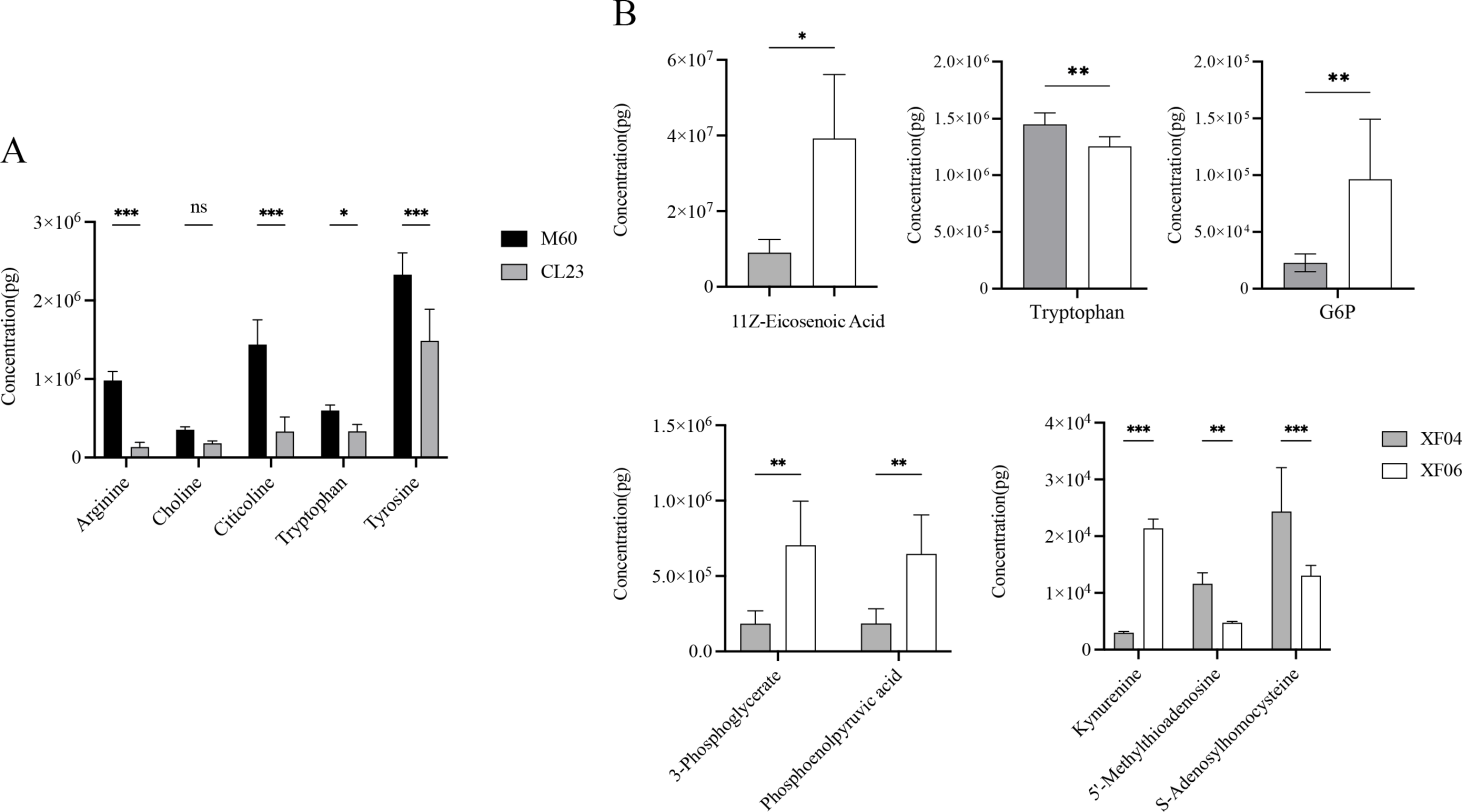
Quantitative analysis of metabolites. (A) The content of Arginine, Choline, Citicoline, Tryptophan and Tryrosine in the M60 and CL23 cell groups. (B) The content of 11Z-Eicosenoic acid, Tryptophan, Glucose 6-phosphate, 3-Phosphoglycerate, phosphoenolpyruvic acid, Kynurenine, 5′-Methylthioadenosine and S-Adenosylhomocysteine in the XF04 and XF06 cell groups. * *p* < 0.05, ** *p* < 0.01, *** *p* < 0.001, ns indicates no significant difference.

The quantitative analysis of the metabolites revealed a decrease in the levels of arginine, choline, citicoline, tryptophan, and tyrosine compared to MDCK-M60 cells (Fig. 6A). Of these, the concentrations of choline, tryptophan, and tyrosine were in accordance with the changing trend observed in the untargeted metabolomics results, while the decreases seen in arginine, citicoline, and glycerol 3-phosphate were contrary to the untargeted metabolomics results. The levels of 5′-methylthioadenosine, S-adenosylhomocysteine, tryptophan, kynurenine, 11Z-eicosenoic acid, 3-phosphoglycerate, Glucose 6-phosphate, and phosphoenolpyruvic acid seen in the quantitative metabolomics analysis were consistent with the untargeted metabolomics results (Fig. 6B). Consistent with the untargeted metabolomics results, the content of 5′-methylthioadenosine, S-adenosylhomocysteine, and tryptophan showed a decrease, and the levels of kynurenine, 11Z-eicosenoic acid, 3-phosphoglycerate, Glucose 6-phosphate, and phosphoenolpyruvic acid showed an increase.

**Figure 7:**
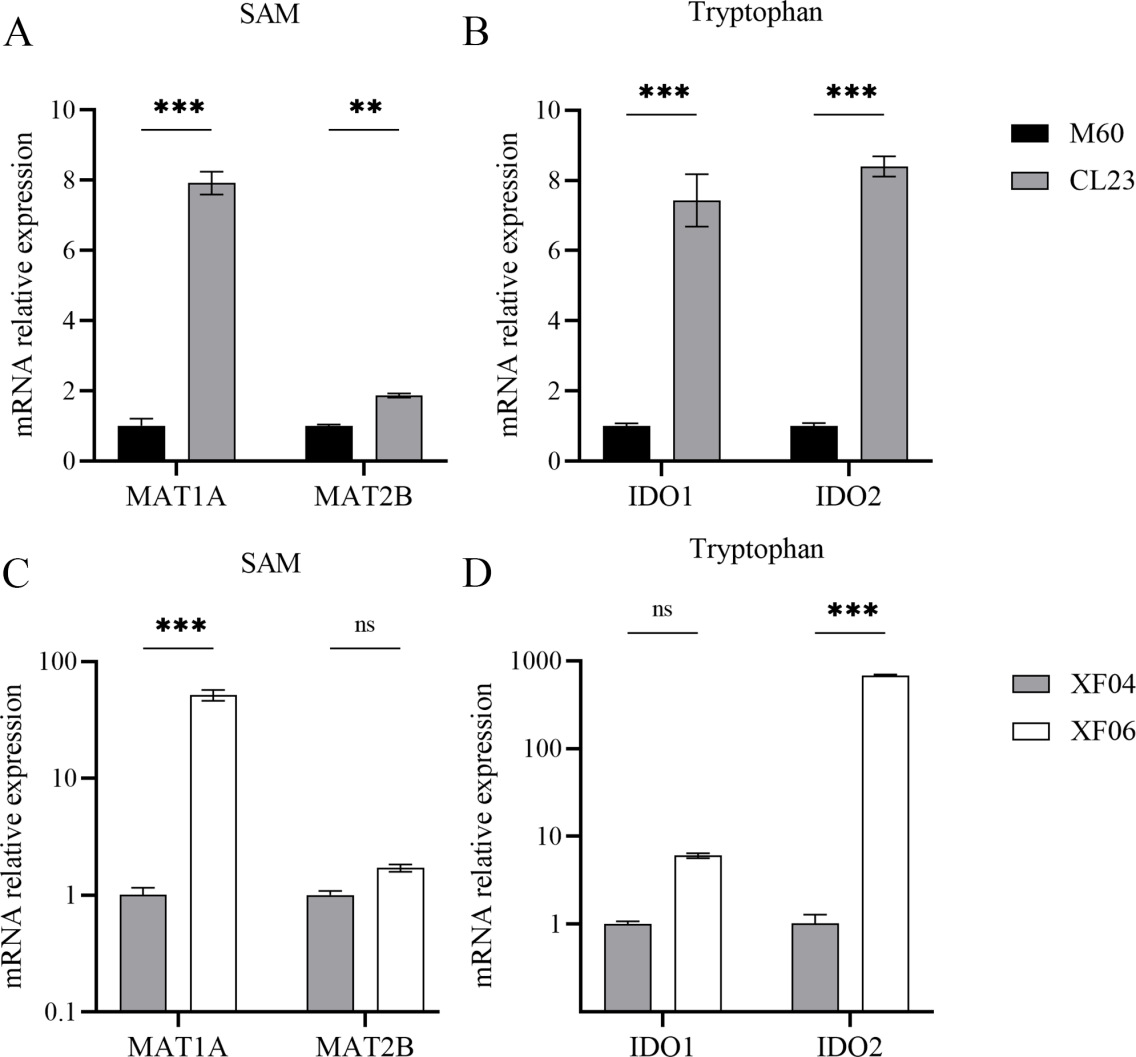
Expression levels of mRNA for metabolic enzymes. (A, B) RT-qPCR analysis of SAM and tryptophan metabolic enzymes in M60 and CL23; and (C, D) in XF04 and XF06. * *p* < 0.05, ** *p* < 0.01, *** *p* < 0.001 ns indicates no significant difference.

The associated metabolites and enzymes were then explored at the mRNA level, and their corresponding genes were identified. The mRNA expression levels of SAM-related enzymes (MAT1A, MAT2B), and tryptophan-related enzymes (IDO1, IDO2) showed a higher increase in the non-tumorigenic cell groups than in the tumorigenic cell groups (Fig. 7). This caused a decrease in tryptophan levels in both the untargeted and targeted metabolomics analyses and an increase in the expression levels of downstream metabolic enzymes. Because SAM and citicoline levels were both increased (Table S2), but choline and tryptophan were both decreased, choline and tryptophan were identified as promoting cell growth.

**Figure 8:**
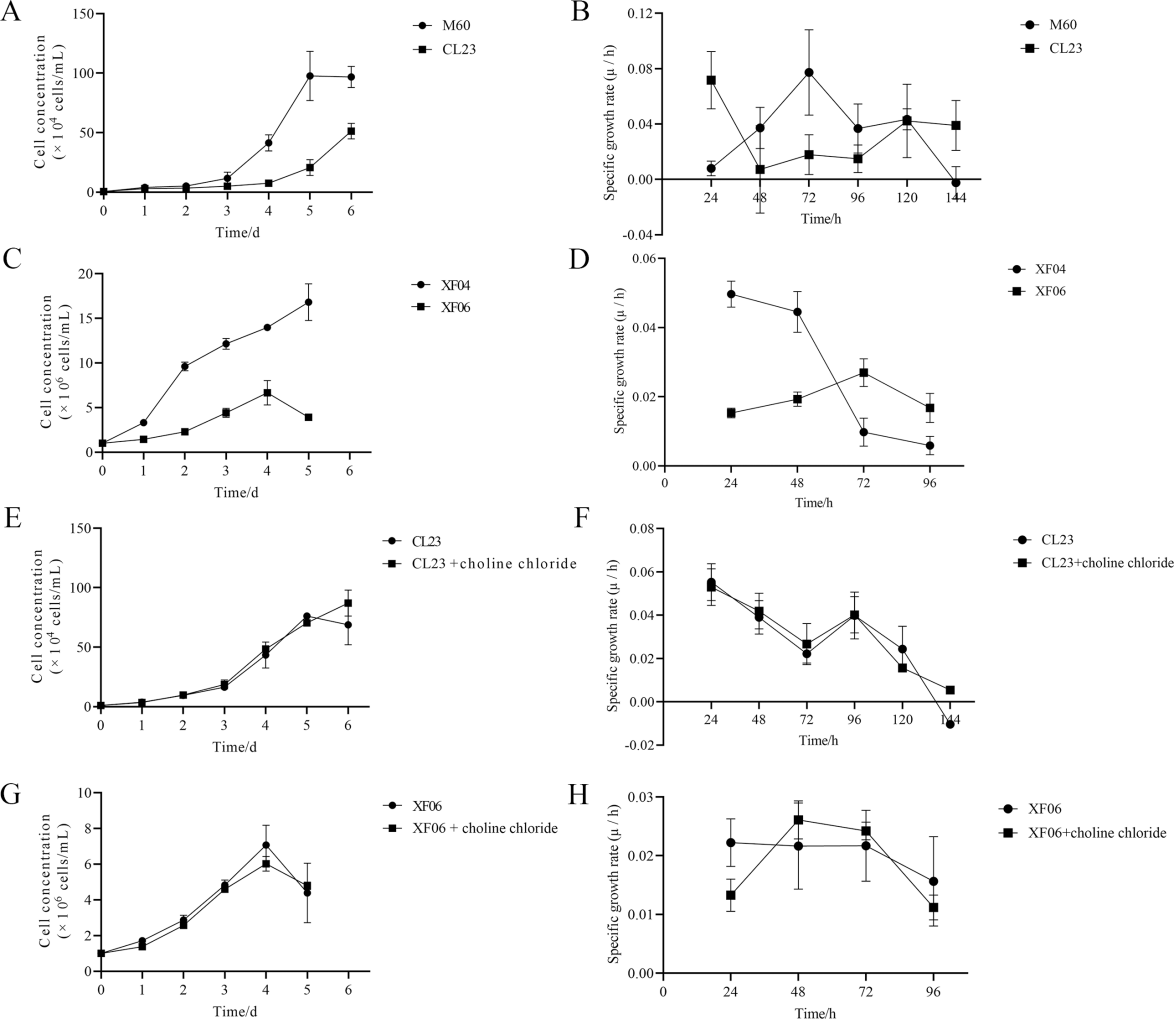
The growth curve and specific growth rate of cells. (A, B) The growth curves and specific growth rates of M60 and CL23 cells; and (C, D) of XF04 and XF06 cells, respectively. (E, F) The growth curves and specific growth rates of M60 and CL23 cells treated with 40 µM choline chloride; and (G, H) of XF04 and XF06 cells treated with 40 µM choline chloride, respectively.

The results of this study show that choline chloride addition had a significant effect on promoting cell viability at 24 and 48 h (Figs. S3A and S3B). However, there were no significant differences on cell viability with tryptophan present (Figs. S3C and S3D), indicating that tryptophan did not impact proliferation rate (cell growth curve) or specific growth rate (µ/h). As shown in Figs. 8A and 8B, the proliferation rate (cell growth curve) of MDCK-CL23 cells was significantly lower than that of MDCK-M60 cells, yet the specific growth rate (µ/h) was only slightly lower than that of M60 cells. The same results were seen in MDCK-XF06 cells compared to MDCK-XF04 cells (Fig. 8C), however, the specific growth rate (µ/h) of XF06 cells was significantly lower than that of XF04 cells (Fig. 8D). Choline chloride (40 µM) was then added to both CL23 and XF06 cells with a 5–6-day cultivation. The data showed that choline chloride addition had a slight promotional effect on the proliferation rate of CL23 (Figs. 8E, 8F) and XF06 cells (Figs. 8G, 8H).

## Discussion

In order to enhance the safety of influenza vaccines, two MDCK cell lines with higher safety coefficients were established in our laboratory: the adherent MDCK-CL23 cell line and the suspension MDCK-XF06 cell line. However, the proliferation rate of these cell groups were slower than that of the control groups, hindering the potential vaccine production cycle. For these two cell lines to be useful, their proliferation rates would need to increase.

In this study, we used both a untargeted and targeted metabolomics approach to help identify the differences between the high and low proliferation rates. The results of the differential metabolite analysis revealed that S-adenosylmethionine and citicoline, both associated with choline metabolism, were significantly upregulated in MDCK-CL23 cells. Choline metabolism has been identified as a key factor in controlling cell multiplication and apoptosis (Ridgway, 2013), so it may also play a role in the growth of MDCK-CL23 cells. Conversely, folic acid, 6-hydroxymelatonin, and D-(-)-3-phosphoglyceric acid were significantly downregulated in the MDCK-CL23 cells. These metabolites are involved in a variety of physiological processes related to cell division, such as folate affecting the proliferation and differentiation of C2C12 myoblasts (Hwang et al., 2018), and melatonin in tryptophan metabolism facilitating differentiation of MSCs cells (Michalowska et al., 2015). In the suspension cell group, L-kynurenine, 5-hydroxyindoleacetate, formylkynurenine, and phosphoenolpyruvic acid were upregulated by more than 20-fold compared to the MDCK-XF04 cell group, and were mainly involved in the tryptophan-kynurenine pathway (Fig. 3B and Table S3). Around 95% of tryptophan is broken down through the kynurenine pathway into cofactor NAD+, which is essential for preventing cellular senescence and for energy production (Dehhaghi, Kazemi Shariat Panahi & Guillemin, 2019). Adenine, guanosine monophosphate, deoxyguanosine monophosphate, and S-adenosylmethionine were significantly downregulated, and were mainly involved in purine metabolism.

Riboflavin metabolism was one of the six significantly altered pathways between the MDCK-M60 and MDCK-CL23 cell groups. Riboflavin is an indispensable water-soluble vitamin, also referred to as vitamin B2, necessary for the production of flavin adenine dinucleotide (FAD) and flavin mononucleotide (FMN). It is involved in many physiological functions and is key to cell growth (Suwannasom et al., 2020). Nakano et al. (2011) found that riboflavin depletion can suppress the proliferation of Caco-2 cells and have long-term effects on cell viability. In addition, β-alanine metabolism is also correlated to the adherent cell lines (M60 and CL23 cells). β-alanine forms a part of the synthetic acetyl-CoA and is a critical factor in regulating glycometabolism, lipid metabolism, and protein metabolism (Fig. 3). Previous studies have also indicated that β-alanine may be associated with cell energy metabolism and proliferation (Comerford et al., 2014; Li et al., 2018; You et al., 2021). The metabolic processing of tryptophan was notably increased in the MDCK-XF06 cell group. Tryptophan and its metabolites (kynurenine, NAD+, 5-HT, *etc*.) are essential for cell growth and cellular homeostasis (Mondanelli, Volpi & Orabona, 2022; Platten et al., 2019). Another enriched pathway was the pentose phosphate pathway, which is a major pathway for glucose catabolism that produces NADPH and ribose 5-phosphate (R5P). This pathway is imperative in sustaining the balance of the cell’s redox state, as it produces phosphopentoses and ribonucleotide, thereby affecting cell proliferation and survival (Ge et al., 2020; Patra & Hay, 2014; Stincone et al., 2015).

Recent research has unveiled a variety of metabolic pathways pertinent to cellular physiology. For example, the riboflavin and tryptophan metabolic pathways in low-proliferated cells were recently found to be significantly altered, implying a potential link to decreased cell growth (Lu et al., 2020; Michalowska et al., 2015). Thus, metabolite addition or metabolic enzymes modification could be applied to explore the mechanisms of this association.

Untargeted metabolomics enables extensive detection of metabolites in samples, while targeted metabolomics provides precise quantification and characterization of metabolites to reduce non-targeted false positives. We utilized high-throughput targeted metabolomics to further validate the presence of potential metabolites. In MDCK-CL23 cells, arginine and citicoline trends were incongruous with the untargeted metabolomics results, indicating a difference between the two techniques for detecting small molecules. However, the remaining compounds followed the same trend as those observed using untargeted metabolomics. The concentrations of choline, tryptophan, and tyrosine were all decreased in MDCK-CL23 cells, suggesting that they play major roles in cell metabolism. Choline is essential for sustaining the normal function and stability of cells (Sanders & Zeisel, 2007). It is a precursor of citicoline and phosphatidylcholine, which has been linked to cell signaling as an inducer of apoptosis (Bernhard, Poets & Franz, 2019). The levels of tryptophan were decreased in both MDCK-CL23 and MDCK-XF06 cells (Tables S2 and S3). Multiple studies have found that the regulation of tryptophan metabolism is essential in the proliferation of human pluripotent stem cells (hPSC) (Cervenka, Agudelo & Ruas, 2017; Someya et al., 2021).

Methionine adenosyltransferase (MAT) is a vital cellular enzyme responsible for the production of S-adenosylmethionine (SAM), a vital biological methyl group (Zhao et al., 2019). MAT is composed of MAT1A and MAT2A, which are mainly distributed in normal liver and extrahepatic tissues, respectively. MAT1A is responsible for encoding MATα1 and maintaining cell differentiation, and MAT2B encodes for the β regulatory subunit, which controls MATII activity (Murray et al., 2019). We demonstrated that MAT1A and MAT2B levels were increased in both cell groups, suggesting a progressive production of SAM (Fig. 7). The concentration of SAM decreased significantly in MDCK-XF06 cells, but there was no significant difference observed in MDCK-CL23 cells. This could be attributed to suspension acclimation. Both IDO1 and IDO2 are the first step rate-limiting enzymes of the tryptophan-kynurenine pathway and are immunosuppressive molecules (Liu et al., 2016; Merlo, Peng & Mandik-Nayak, 2022). The elevated expressions of IDO1 and IDO2 observed in this study indicate a high consumption of tryptophan, which is consistent with the low levels of tryptophan observed in both cells.

Choline is a precursor of cell membrane phospholipids and acetylcholine, which can be metabolized into betaine and subsequently converted into SAM, serving as a methyl group donor. Choline metabolism is closely related to cell proliferation and apoptosis (Zeisel, 1990), and a lack of choline can inhibit the proliferation of neuroblastoma cells and the proliferation of hippocampal neural epithelial stem cells in rat and mouse embryos (Albright et al., 1999; Niculescu, Yamamuro & Zeisel, 2004). Based on the results of this study, the exogenous addition of choline chloride may not have a significant effect on cell proliferation. Therefore, an approach that originally modificates the differential genes related with choline metabolism may be promising. Modification with related metabolic enzymes could also help to promote cell proliferation.

## Conclusions

Using untargeted and targeted metabolomics, significant differences in changes in choline metabolism and tryptophan metabolism were observed between the tumorigenic and non-tumorigenic MDCK cells. Cell proliferation experiments showed that adding additional choline or tryptophan (Figs. S3E and S3F) did not effectively increase the cell proliferation rate. RT-qPCR results found that MAT1A, IDO1, and IDO2 were extremely upregulated, indicating a mRNA-level modification of metabolic enzymes could be optimized.

## Supplemental Information

10.7717/peerj.16077/supp-1 Supplemental Information 1 Score plot of PCA among four groups of cells in targeted metabolomics

10.7717/peerj.16077/supp-2 Supplemental Information 2 Permutation tests of OPLS-DA models for CL23 *vs* M60 (A) and XF06 *vs* XF04 (B)

10.7717/peerj.16077/supp-3 Supplemental Information 3 Cell viability and proliferation (A) The viability of CL23 cells was significantly promoted by different concentrations of choline chloride for 24 h; and 48 h (B); (C) The viability of CL23 cells was slightly promoted by different concentrations of tryptophan for 24 h; and 48 h (D); (E) The growth curves and specific growth rates (F) of XF06 cells and XF06 added with tryptophan. *p < 0.05, **p < 0.01, and ***p < 0.001.

10.7717/peerj.16077/supp-4 Supplemental Information 4 Description of primers

10.7717/peerj.16077/supp-5 Supplemental Information 5 Differential metabolites of MDCK-CL23 *vs* MDCK-M60 cell groups

10.7717/peerj.16077/supp-6 Supplemental Information 6 Differential metabolites of MDCK-XF06 *vs* MDCK-XF04 cell groups

10.7717/peerj.16077/supp-7 Supplemental Information 7 Raw data of RT-qPCR

10.7717/peerj.16077/supp-8 Supplemental Information 8 Raw Data: untargeted metabolomics

10.7717/peerj.16077/supp-9 Supplemental Information 9 Raw Data: targeted metabolomics

10.7717/peerj.16077/supp-10 Supplemental Information 10 Raw data of Fig. 8

10.7717/peerj.16077/supp-11 Supplemental Information 11 Raw data of Fig. S3

## Additional Information and Declarations

### Data Availability

The following information was supplied regarding data availability: The raw data is available in the Supplemental Files.
