# Supplementary figures and images for: Metabolomics profiling reveals differences in proliferation between tumorigenic and non-tumorigenic Madin-Darby canine kidney (MDCK) cells
Source: PeerJ. 2023 Sep 20;11:e16077. doi: 10.7717/peerj.16077 (PMC10517658; doi:10.7717/peerj.16077)

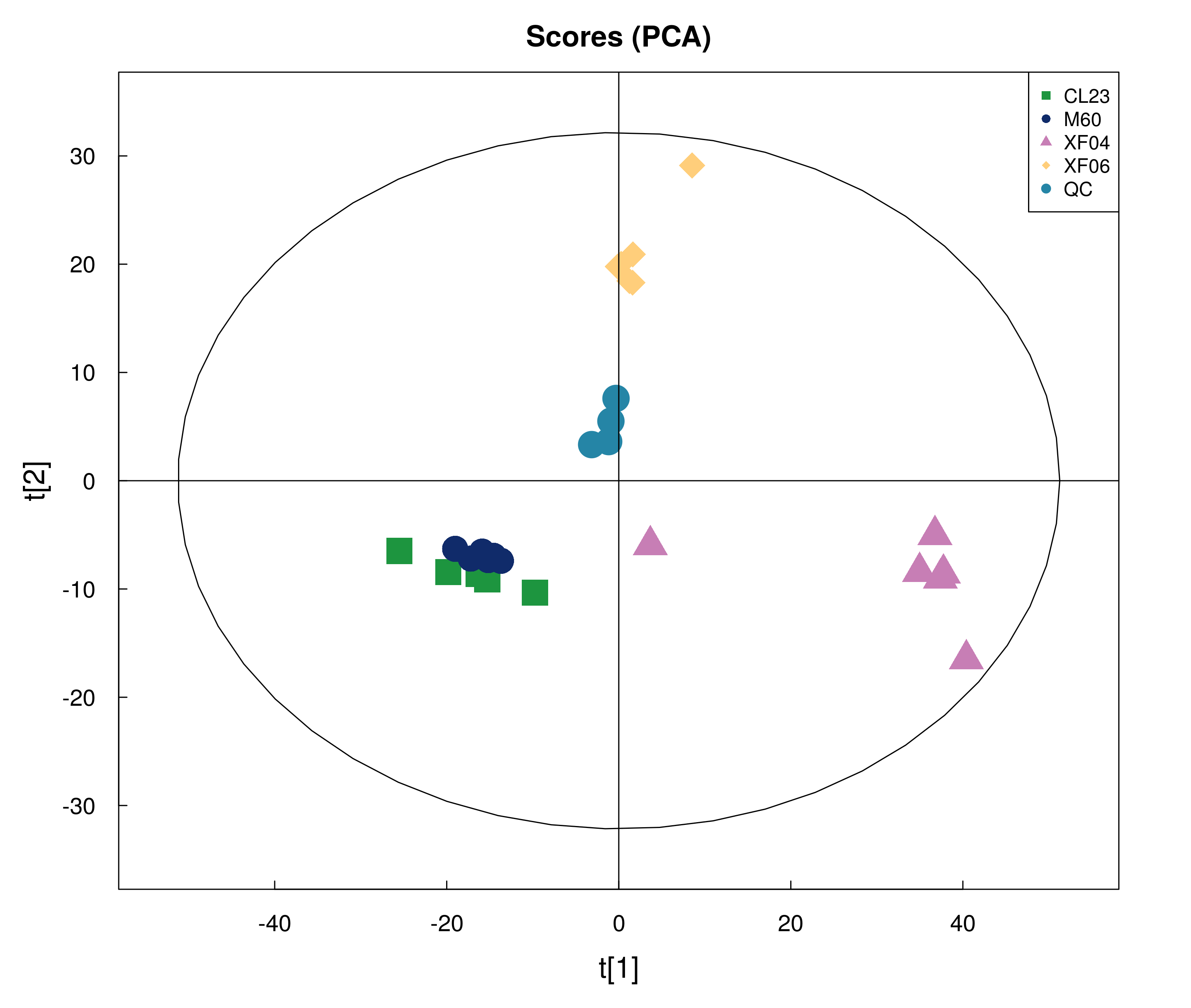

Supplement: Supplemental Information 1 [file peerj-11-16077-s001.png]

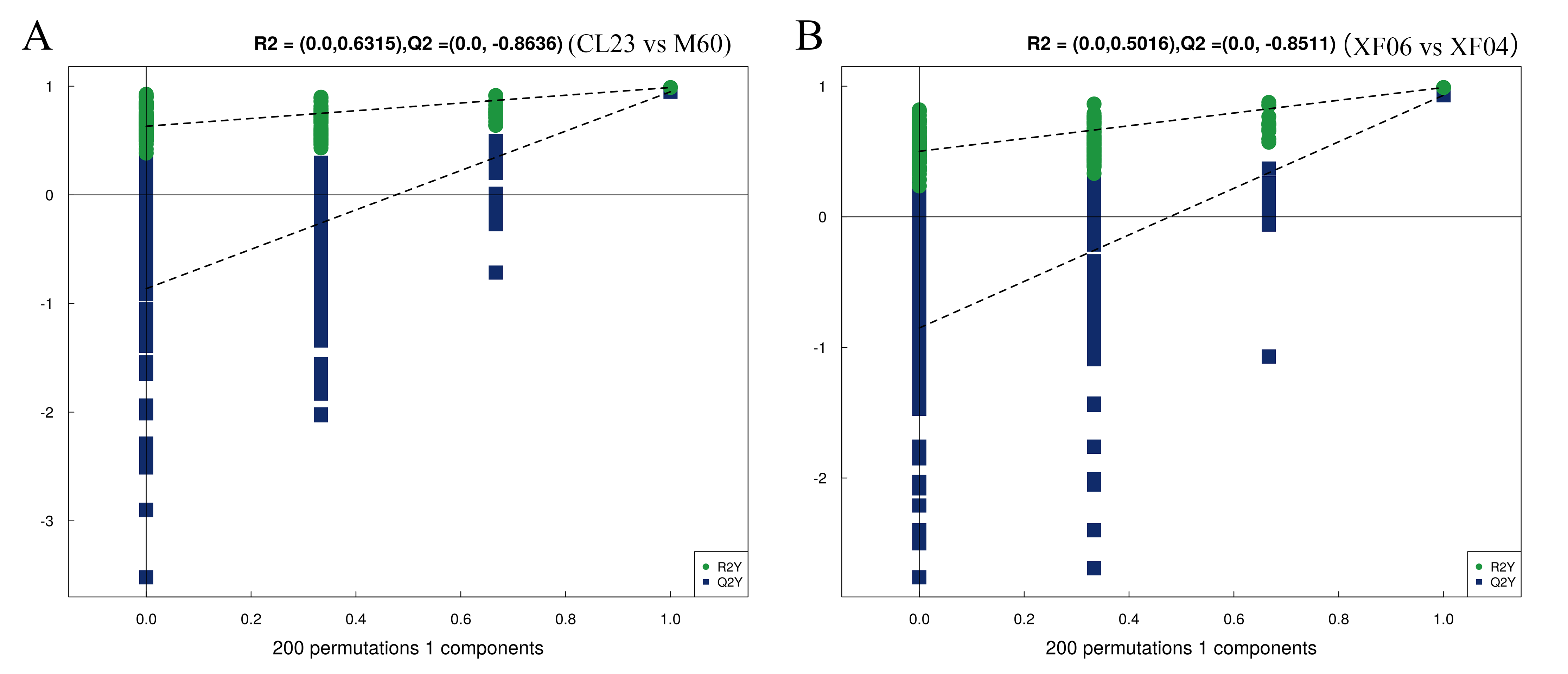

Supplement: Supplemental Information 2 [file peerj-11-16077-s002.png]

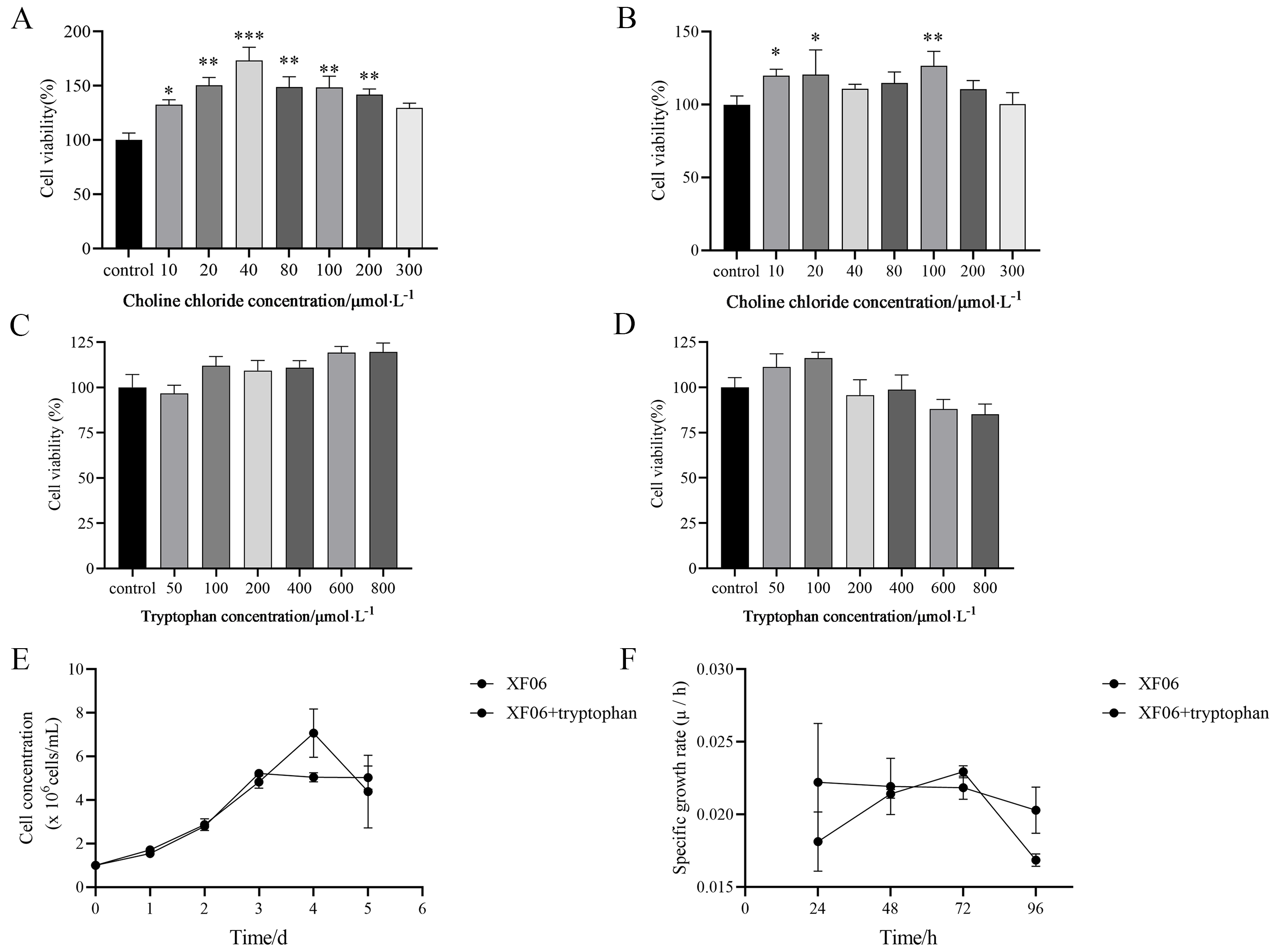

Supplement: Supplemental Information 3 — (A) The viability of CL23 cells was significantly promoted by different concentrations of choline chloride for 24 h; and 48 h (B); (C) The viability of CL23 cells was slightly promoted by different concentrations of tryptophan for 24 h; and 48 h (D); (E) The growth curves and specific growth rates (F) of XF06 cells and XF06 added with tryptophan. *p < 0.05, **p < 0.01, and ***p < 0.001. [file peerj-11-16077-s003.png]
