# Supplementary material for: Metabolomics profiling reveals differences in proliferation between tumorigenic and non-tumorigenic Madin-Darby canine kidney (MDCK) cells
Source: PeerJ. 2023 Sep 20;11:e16077. doi: 10.7717/peerj.16077 (PMC10517658; doi:10.7717/peerj.16077)
Supplement: Supplemental Information 4 [file peerj-11-16077-s004.docx]

Table S1. Description of primers

| Gene | Forward sequence | Reverse sequence |
| --- | --- | --- |
| GAPDH | TCCGATGCCTGCTTCACTAC | TGCCCAGAACATCATCCCTG |
| MATIA | GCTTTCAGAATGCGTGGGTG | AGAAGCCATCTGGTGAAGCC |
| MAT2B | GAACGAGCTCTCCATCCACT | CCCAGTGGCACCAGTAATCA |
| IDO1 | GCTTTGACATTCTGCTGGGC | CGAACAAACTCCCGGACTGA |
| IDO2 | TGGACACTCAGGAACTCAGA | TGGCAATTTCCATCCAAGGC |
