# Supplementary material for: Metabolomics profiling reveals differences in proliferation between tumorigenic and non-tumorigenic Madin-Darby canine kidney (MDCK) cells
Source: PeerJ. 2023 Sep 20;11:e16077. doi: 10.7717/peerj.16077 (PMC10517658; doi:10.7717/peerj.16077)
Supplement: Supplemental Information 5 [file peerj-11-16077-s005.doc]

Table S2. Differential metabolites of MDCK-CL23 vs MDCK-M60 cell groups.

| Mode | NO. | Name | KEGG ID | VIP | Fold change | p.value | Pathway | Trend |
| --- | --- | --- | --- | --- | --- | --- | --- | --- |
| Positive Mode | 1 | S-adenosylmethionine | C00019 | 2.4301 | 30.1545 | 0.0018 | Cysteine and methionine metabolism; Arginine and proline metabolism; Biosynthesis of amino acids | up |
|  | 2 | Citicoline | C00307 | 2.6242 | 20.755 | 0.0003 | Glycerophospholipid metabolism; Choline metabolism in cancer | up |
|  | 3 | Nicotinamide adenine dinucleotide (nad+) | C00003 | 1.8927 | 8.2575 | 0 | Oxidative phosphorylation; Nicotinate and nicotinamide metabolism; AMPK signaling pathway | up |
|  | 4 | Sn-glycerol 3-phosphate | C00093 | 1.5575 | 7.4481 | 0.0028 | Glycerolipid metabolism; Glycerophospholipid metabolism; ABC transporters; Choline metabolism in cancer | up |
|  | 5 | (1e)-n-hydroxy-4-(methylsulfanyl)-1-butanimine | C17241 | 1.4197 | 5.9085 | 0 | 2-Oxocarboxylic acid metabolism | up |
|  | 6 | Diethanolamine | C06772 | 2.0278 | 5.53 | 0.0015 | Glycerophospholipid metabolism | up |
|  | 7 | 2-(hydroxyamino)-1-methyl-6-phenylimidazo[4,5-b]pyridine | C20286 | 1.5157 | 5.3864 | 0.0064 | Chemical carcinogenesis | up |
|  | 8 | N-methylethanolamine phosphate | C01210 | 1.3332 | 5.2603 | 0.0096 | Glycerophospholipid metabolism | up |
|  | 9 | 4-(methylnitrosamino)-1-(3-pyridyl)-1-butanol glucuronide | C19605 | 1.4849 | 5.0006 | 0.0044 | Metabolism of xenobiotics by cytochrome P450 | up |
|  | 10 | Cholecalciferol | C05443 | 1.7448 | 4.7733 | 0.0001 | Steroid biosynthesis; Vitamin digestion and absorption | up |
|  | 11 | Dichloroacetic acid | C11149 | 2.0751 | 4.2071 | 0.012 | Metabolism of xenobiotics by cytochrome P450 | up |
|  | 12 | Biotin sulfone | C20387 | 1.4303 | 3.7137 | 0.0018 | Biotin metabolism | up |
|  | 13 | Sphinganine | C00836 | 1.5537 | 3.6641 | 0 | Sphingolipid metabolism; Sphingolipid signaling pathway | up |
|  | 14 | Î±-n-acetylcitrulline | C15532 | 1.616 | 3.1947 | 0.0036 | Arginine biosynthesis; Biosynthesis of amino acids | up |
|  | 15 | 4-hydroxy-3-octaprenylbenzoic acid | C05809 | 1.1306 | 2.908 | 0 | Ubiquinone and other terpenoid-quinone biosynthesis | up |
|  | 16 | B-ala-lys | C05341 | 1.2839 | 2.7331 | 0.0112 | beta-Alanine metabolism | up |
|  | 17 | Tyrosol | C06044 | 1.3787 | 2.4603 | 0.0012 | Tyrosine metabolism | up |
|  | 18 | L-arginine | C00062 | 1.5676 | 2.3648 | 0.0038 | Aminoacyl-tRNA biosynthesis; Biosynthesis of amino acids; mTOR signaling pathway; Central carbon metabolism in cancer | up |
|  | 19 | Phthalic acid | C01606 | 1.1619 | 2.0699 | 0.0097 | ABC transporters; | up |
|  | 20 | L-methionine | C00073 | 1.2352 | 0.4911 | 0.004 | Cysteine and methionine metabolism; Aminoacyl-tRNA biosynthesis; Central carbon metabolism in cancer | down |
|  | 21 | Biotin l-sulfoxide | C20386 | 1.1321 | 0.4214 | 0.0018 | Biotin metabolism | down |
|  | 22 | L-carnitine | C00318 | 1.3533 | 0.4206 | 0.0028 | Thermogenesis; Bile secretion | down |
|  | 23 | Choline | C00114 | 1.2448 | 0.4178 | 0 | Glycine, serine and threonine metabolism; Glycerophospholipid metabolism; Choline metabolism in cancer | down |
|  | 24 | Cytosine | C00380 | 1.2566 | 0.4113 | 0.0123 | Pyrimidine metabolism | down |
|  | 25 | Deoxyguanosine monophosphate | C00362 | 1.1083 | 0.4051 | 0.0118 | Purine metabolism | down |
|  | 26 | 2-hydroxycinnamic acid | C01772 | 1.0692 | 0.3963 | 0.0197 | Phenylalanine metabolism | down |
|  | 27 | Benzene | C01407 | 1.3981 | 0.3377 | 0 | Metabolic pathways | down |
|  | 28 | Glycerin | C00116 | 1.6992 | 0.3288 | 0.0004 | Galactose metabolism; Glycerolipid metabolism | down |
|  | 29 | Gamma-linolenic acid | C06426 | 1.2863 | 0.3072 | 0 | Linoleic acid metabolism; Biosynthesis of unsaturated fatty acids | down |
|  | 30 | Indole-3-acetamide | C02693 | 1.019 | 0.3049 | 0.0173 | Tryptophan metabolism | down |
|  | 31 | Pantothenic acid | C00864 | 1.4484 | 0.2849 | 0.0009 | beta-Alanine metabolism; Pantothenate and CoA biosynthesis; Vitamin digestion and absorption | down |
|  | 32 | 16-hydroxyhexadecanoic acid | C18218 | 1.7806 | 0.2492 | 0 | Metabolic pathways | down |
|  | 33 | 4-o-(2-acetamido-2-deoxy-beta-d-glucopyranosyl)-beta-d-glucopyranuronic acid | C00518 | 1.5475 | 0.2481 | 0.0001 | Fluid shear stress and atherosclerosis | down |
|  | 34 | 4-acetamidobutanoate | C02946 | 1.2017 | 0.2439 | 0.0015 | Arginine and proline metabolism | down |
|  | 35 | Palmitoleic acid | C08362 | 1.8063 | 0.2427 | 0 | Fatty acid biosynthesis | down |
|  | 36 | Retinoate | C00777 | 1.1644 | 0.2375 | 0 | Retinol metabolism; Th17 cell differentiation; Pathways in cancer | down |
|  | 37 | 1-nitrosonaphthalene | C14788 | 1.4042 | 0.2345 | 0.0062 | Metabolism of xenobiotics by cytochrome P450 | down |
|  | 38 | N-acetylserotonin | C00978 | 2.0101 | 0.2269 | 0 | Tryptophan metabolism | down |
|  | 39 | L-tryptophan | C00078 | 1.4065 | 0.2251 | 0.0016 | Aminoacyl-tRNA biosynthesis; Biosynthesis of amino acids; Central carbon metabolism in cancer | down |
|  | 40 | 7-aminomethyl-7-deazaguanine | C16675 | 1.1496 | 0.2079 | 0.0083 | Folate biosynthesis | down |
|  | 41 | Adenine | C00147 | 1.7422 | 0.2038 | 0.0047 | Purine metabolism | down |
|  | 42 | 1,2-dehydroreticuline | C06167 | 2.2275 | 0.1651 | 0 | Metabolic pathways | down |
|  | 43 | Ajmalicine | C09024 | 1.7075 | 0.1575 | 0 | Metabolic pathways | down |
|  | 44 | D-(-)-3-phosphoglyceric acid | C00197 | 2.3372 | 0.1358 | 0 | Glycerolipid metabolism; Carbon metabolism; Biosynthesis of amino acids | down |
|  | 45 | Dopamine | C03758 | 2.4064 | 0.1042 | 0 | Tyrosine metabolism; cAMP signaling pathway | down |
|  | 46 | Folic acid | C00504 | 2.5832 | 0.086 | 0 | One carbon pool by folate; Folate biosynthesis; Vitamin digestion and absorption | down |
|  | 47 | (6r)-l-erythro-6,7-dihydrobiopterin | C00268 | 2.1804 | 0.0801 | 0.0006 | Folate biosynthesis | down |
|  | 48 | 6-hydroxymelatonin | C05643 | 3.3535 | 0.0321 | 0 | Tryptophan metabolism | down |
| Negative Mode | 49 | (2e,6e)-farnesyl monophosphate | C20121 | 1.3324 | 8.9691 | 0.0012 | Terpenoid backbone biosynthesis | up |
|  | 50 | Cyclic adp-ribose | C13050 | 1.8611 | 8.3601 | 0 | Calcium signaling pathway | up |
|  | 51 | Xanthine | C00385 | 1.5307 | 8.3092 | 0.0001 | Purine metabolism | up |
|  | 52 | Mhpg | C05594 | 1.3783 | 6.3238 | 0.0046 | Tyrosine metabolism | up |
|  | 53 | Uridine 5'-diphosphogalactose | C00052 | 1.9322 | 5.8137 | 0 | Galactose metabolism; Amino sugar and nucleotide sugar metabolism | up |
|  | 54 | Succinate | C00042 | 1.7406 | 3.6024 | 0 | Citrate cycle (TCA cycle); Tyrosine metabolism; Pyruvate metabolism; Carbon metabolism; cAMP signaling pathway | up |
|  | 55 | Uridine 5'-triphosphate | C00075 | 1.3876 | 3.4273 | 0.0001 | Pyrimidine metabolism | up |
|  | 56 | L-threonic acid | C01620 | 1.3739 | 3.1143 | 0 | Ascorbate and aldarate metabolism | up |
|  | 57 | Pyrophosphoric acid | C00013 | 1.2475 | 2.9913 | 0 | Oxidative phosphorylation | up |
|  | 58 | Cysteine-glutathione disulfide | C05526 | 1.1244 | 2.5464 | 0.003 | Cysteine and methionine metabolism | up |
|  | 59 | Uridine 5'-diphosphoglucuronic acid | C00167 | 1.3933 | 2.5241 | 0.0004 | Amino sugar and nucleotide sugar metabolism | up |
|  | 60 | 16-hydroxyhexadecanoic acid | C18218 | 1.2828 | 2.5038 | 0 | Metabolic pathways | up |
|  | 61 | Hexadecanedioic acid | C19615 | 1.0697 | 2.4785 | 0 | Metabolic pathways | up |
|  | 62 | Galacturonate 1-phosphate | C04037 | 1.106 | 0.5046 | 0.0015 | Amino sugar and nucleotide sugar metabolism | down |
|  | 63 | L-tyrosine | C00082 | 1.0024 | 0.4881 | 0.0009 | Aminoacyl-tRNA biosynthesis; Biosynthesis of amino acids; Central carbon metabolism in cancer | down |
|  | 64 | L-(-)-methionine | C00073 | 1.0822 | 0.4741 | 0.0001 | Aminoacyl-tRNA biosynthesis; Biosynthesis of amino acids; Central carbon metabolism in cancer | down |
|  | 65 | Ribulose diphosphate | C01182 | 1.2122 | 0.4294 | 0.0004 | Glyoxylate and dicarboxylate metabolism; Carbon metabolism | down |
|  | 66 | Palmitoleic acid | C08362 | 1.3375 | 0.4267 | 0 | Fatty acid biosynthesis | down |
|  | 67 | L-threonine | C00188 | 1.1131 | 0.3747 | 0 | Glycine, serine and threonine metabolism; Valine, leucine and isoleucine biosynthesis; ABC transporters | down |
|  | 68 | Deoxycytidine diphosphate | C00705 | 1.1137 | 0.3702 | 0.0014 | Pyrimidine metabolism | down |
|  | 69 | Riboflavin | C00255 | 1.225 | 0.3586 | 0 | Riboflavin metabolism; ABC transporters | down |
|  | 70 | L-proline | C00148 | 1.0264 | 0.3534 | 0 | Arginine and proline metabolism; Aminoacyl-tRNA biosynthesis; ABC transporters; Central carbon metabolism in cancer | down |
|  | 71 | Flavin mononucleotide (fmn) | C00061 | 1.4851 | 0.3432 | 0.0001 | Oxidative phosphorylation; Riboflavin metabolism | down |
|  | 72 | L-(+)-lactic acid | C00186 | 1.4094 | 0.29 | 0 | Glycolysis / Gluconeogenesis; Pyruvate metabolism; cAMP signaling pathway; HIF-1 signaling pathway; Central carbon metabolism in cancer | down |
|  | 73 | 3'-dephosphocoenzyme a | C00882 | 1.1728 | 0.2776 | 0.0058 | Pantothenate and CoA biosynthesis | down |
|  | 74 | D-(-)-mannitol | C00392 | 1.3064 | 0.2655 | 0 | Fructose and mannose metabolism; ABC transporters | down |
|  | 75 | Taxifolin | C01617 | 1.5694 | 0.2522 | 0.0018 | Metabolic pathways | down |
|  | 76 | Fmet | C03145 | 1.1174 | 0.245 | 0.0036 | Cysteine and methionine metabolism | down |
|  | 77 | Rosmarinic acid | C01850 | 1.4981 | 0.2353 | 0.0002 | Tyrosine metabolism | down |
|  | 78 | 20-hydroxy-(5z,8z,11z,14z)-eicosatetraenoic acid | C14748 | 1.3218 | 0.2258 | 0.0008 | Arachidonic acid metabolism | down |
|  | 79 | Uracil | C00106 | 1.5215 | 0.1716 | 0.0056 | Pyrimidine metabolism; beta-Alanine metabolism; Pantothenate and CoA biosynthesis | down |
|  | 80 | (+/-)-pantetheine | C00831 | 1.1586 | 0.1549 | 0.0032 | Pantothenate and CoA biosynthesis | down |
|  | 81 | Deoxyguanosine monophosphate | C00362 | 1.3595 | 0.1535 | 0.0003 | Purine metabolism | down |
|  | 82 | Adenine | C00147 | 1.8195 | 0.1331 | 0.0001 | Purine metabolism | down |
|  | 83 | Cytidine | C00475 | 1.9466 | 0.1194 | 0 | Pyrimidine metabolism; ABC transporters; | down |
|  | 84 | Adenylsuccinic acid | C03794 | 1.8761 | 0.1161 | 0 | Purine metabolism; Alanine, aspartate and glutamate metabolism | down |
|  | 85 | beta-alanine | C00099 | 2.0074 | 0.098 | 0.0005 | Pyrimidine metabolism; beta-Alanine metabolism; Pantothenate and CoA biosynthesis | down |
|  | 86 | Methotrexate | C01937 | 3.8882 | 0.0164 | 0 | Bile secretion | down |
