# Supplementary material for: Metabolomics profiling reveals differences in proliferation between tumorigenic and non-tumorigenic Madin-Darby canine kidney (MDCK) cells
Source: PeerJ. 2023 Sep 20;11:e16077. doi: 10.7717/peerj.16077 (PMC10517658; doi:10.7717/peerj.16077)
Supplement: Supplemental Information 6 [file peerj-11-16077-s006.doc]

Table S3. Differential metabolites of MDCK-XF06 vs MDCK-XF04 cell groups.

| Mode | NO. | Name | KEGG ID | VIP | Fold change | p.value | Pathway | Trend |
| --- | --- | --- | --- | --- | --- | --- | --- | --- |
| Positive Mode | 1 | L-kynurenine | C00328 | 1.9415 | 33.9584 | 0 | Tryptophan metabolism | up |
|  | 2 | 5-hydroxyindoleacetate | C05635 | 1.949 | 32.4658 | 0 | Tryptophan metabolism | up |
|  | 3 | Formylkynurenine | C02700 | 2.0125 | 28.9305 | 0 | Tryptophan metabolism | up |
|  | 4 | N-succinyl-l-diaminopimelic acid | C04421 | 2.1947 | 20.2452 | 0 | Biosynthesis of amino acids | up |
|  | 5 | Dichloroacetic acid | C11149 | 1.3395 | 19.6664 | 0.0029 | Metabolism of xenobiotics by cytochrome P450 | up |
|  | 6 | Cysteine-glutathione disulfide | C05526 | 2.3247 | 18.9994 | 0 | Cysteine and methionine metabolism | up |
|  | 7 | (+/-)-eucalyptol | C09844 | 1.8575 | 16.6359 | 0 | Inflammatory mediator regulation of TRP channels | up |
|  | 8 | Diethanolamine | C06772 | 1.5674 | 14.2757 | 0.0002 | Glycerophospholipid metabolism | up |
|  | 9 | 3-tropanol | C00729 | 1.3326 | 13.0883 | 0.0035 | Metabolic pathways | up |
|  | 10 | L-citrulline | C00327 | 1.3431 | 10.9764 | 0 | Arginine biosynthesis; Biosynthesis of amino acids; | up |
|  | 11 | L-cysteine | C00097 | 1.3603 | 10.3204 | 0 | Glycine, serine and threonine metabolism; Cysteine and methionine metabolism; Glutathione metabolism; Pantothenate and CoA biosynthesis; Carbon metabolism | up |
|  | 12 | D-(-)-3-phosphoglyceric acid | C00197 | 1.4557 | 9.1096 | 0 | Glycine, serine and threonine metabolism; Cysteine and methionine metabolism; Glycerolipid metabolism; Carbon metabolism | up |
|  | 13 | 4-hydroxy-2-quinolinecarboxylic acid | C01717 | 1.3757 | 8.6412 | 0 | Tryptophan metabolism | up |
|  | 14 | 4-hydroxytamoxifen | C05011 | 1.2561 | 6.8229 | 0.0002 | Drug metabolism - cytochrome P450 | up |
|  | 15 | Υ-glutamylcysteine | C00669 | 1.1437 | 6.6095 | 0 | Glutathione metabolism; Metabolic pathways; Ferroptosis; | up |
|  | 16 | (z)-endoxifen | C16547 | 1.3067 | 6.1766 | 0.0002 | Drug metabolism - cytochrome P450 | up |
|  | 17 | 4-hydroxy-3-octaprenylbenzoic acid | C05809 | 1.2746 | 5.7662 | 0 | Ubiquinone and other terpenoid-quinone biosynthesis | up |
|  | 18 | Dmba | C19488 | 1.0733 | 4.916 | 0 | Metabolism of xenobiotics by cytochrome P450 | up |
|  | 19 | Erythrose, 4-phosphate | C00279 | 1.2392 | 4.7297 | 0 | Pentose phosphate pathway; Phenylalanine, tyrosine and tryptophan biosynthesis; Vitamin B6 metabolism | up |
|  | 20 | 4-(methylnitrosamino)-1-(3-pyridyl-n-oxide)-1-butanol | C19603 | 1.0777 | 4.2418 | 0.0009 | Metabolism of xenobiotics by cytochrome P450 | up |
|  | 21 | L-carnitine | C00318 | 1.2783 | 3.9587 | 0 | Thermogenesis | up |
|  | 22 | Myristic acid | C06424 | 1.3855 | 3.6371 | 0 | Fatty acid biosynthesis | up |
|  | 23 | Dihydroxyindole | C05578 | 1.227 | 2.7845 | 0.0058 | Tyrosine metabolism | up |
|  | 24 | Glutaric acid | C00489 | 1.0027 | 0.3417 | 0.0014 | Fatty acid degradation; Lysine degradation | down |
|  | 25 | N-acetylserotonin | C00978 | 1.1335 | 0.3003 | 0 | Tryptophan metabolism | down |
|  | 26 | (e)-p-coumaric acid | C00811 | 1.4538 | 0.268 | 0 | Ubiquinone and other terpenoid-quinone biosynthesis; Tyrosine metabolism | down |
|  | 27 | Linamarin | C01594 | 1.0558 | 0.2642 | 0 | Metabolic pathways | down |
|  | 28 | Protoporphyrin ix | C02191 | 1.1592 | 0.2215 | 0 | Porphyrin and chlorophyll metabolism | down |
|  | 29 | Thromboxane b2 | C05963 | 1.0389 | 0.2213 | 0 | Arachidonic acid metabolism | down |
|  | 30 | N-methylethanolamine phosphate | C01210 | 1.0685 | 0.2168 | 0 | Glycerophospholipid metabolism | down |
|  | 31 | Uridine 5'-monophosphate | C00105 | 1.3152 | 0.215 | 0 | Pyrimidine metabolism | down |
|  | 32 | S-lactoylglutathione | C03451 | 1.4709 | 0.205 | 0 | Pyruvate metabolism | down |
|  | 33 | L-serine | C00065 | 1.3353 | 0.1935 | 0.0017 | Glycine, serine and threonine metabolism; Cysteine and methionine metabolism; Sphingolipid metabolism; Aminoacyl-tRNA biosynthesis; Carbon metabolism | down |
|  | 34 | 10-hydroxydihydrosanguinarine | C05247 | 1.3364 | 0.1744 | 0 | Metabolic pathways | down |
|  | 35 | Putrescine | C00134 | 1.6579 | 0.1658 | 0 | Arginine and proline metabolism; D-Arginine and D-ornithine metabolism; Glutathione metabolism | down |
|  | 36 | Zalcitabine | C07207 | 1.3964 | 0.1643 | 0.0005 | Bile secretion | down |
|  | 37 | Skatole | C08313 | 1.4016 | 0.1558 | 0 | Tryptophan metabolism | down |
|  | 38 | Eslicarbazepine | C07493 | 1.4717 | 0.1445 | 0 | Drug metabolism - cytochrome P450 | down |
|  | 39 | Folic acid | C00504 | 1.4774 | 0.1404 | 0 | One carbon pool by folate; Folate biosynthesis | down |
|  | 40 | Adenine | C00147 | 1.6657 | 0.1312 | 0 | Purine metabolism | down |
|  | 41 | Formyl-l-methionyl peptide | C03145 | 1.5473 | 0.1291 | 0 | Cysteine and methionine metabolism | down |
|  | 42 | Adenosine 3',5'-diphosphate | C00054 | 1.5469 | 0.1273 | 0.0003 | Purine metabolism; Pantothenate and CoA biosynthesis | down |
|  | 43 | N-acetyl-l-phenylalanine | C03519 | 1.5518 | 0.1263 | 0 | Phenylalanine metabolism | down |
|  | 44 | 16-hydroxyhexadecanoic acid | C18218 | 1.909 | 0.1128 | 0 | Metabolic pathways | down |
|  | 45 | Palmitoleic acid | C08362 | 1.8991 | 0.1128 | 0 | Fatty acid biosynthesis | down |
|  | 46 | (1e)-n-hydroxy-4-(methylsulfanyl)-1-butanimine | C17241 | 1.0587 | 0.1096 | 0.0013 | 2-Oxocarboxylic acid metabolism | down |
|  | 47 | 1,2-dehydroreticuline | C06167 | 1.8737 | 0.1079 | 0 | Metabolic pathways | down |
|  | 48 | Spermine | C00750 | 2.0721 | 0.1046 | 0.0001 | Arginine and proline metabolism; beta-Alanine metabolism; Glutathione metabolism | down |
|  | 49 | L-3-hydroxykynurenine | C03227 | 1.8403 | 0.0937 | 0.0001 | Tryptophan metabolism | down |
|  | 50 | (e)-5-(methylsulfanyl)pentanal oxime | C17245 | 1.6112 | 0.0849 | 0 | 2-Oxocarboxylic acid metabolism | down |
|  | 51 | 5'-methylthioadenosine | C00170 | 1.6715 | 0.0829 | 0 | Cysteine and methionine metabolism | down |
|  | 52 | Xanthosine | C01762 | 1.3039 | 0.077 | 0 | Purine metabolism; ABC transporters; | down |
|  | 53 | Biotin | C00120 | 1.5741 | 0.077 | 0 | Biotin metabolism; ABC transporters; Vitamin digestion and absorption | down |
|  | 54 | (+)-10-deoxymethynolide | C11993 | 2.1869 | 0.0667 | 0 | Metabolic pathways | down |
|  | 55 | Spermidine | C00315 | 1.839 | 0.0643 | 0 | Arginine and proline metabolism; beta-Alanine metabolism; Glutathione metabolism | down |
|  | 56 | Indole-3-acetaldehyde | C00637 | 1.9509 | 0.0643 | 0 | Tryptophan metabolism | down |
|  | 57 | 3-methoxytyramine | C05587 | 2.0172 | 0.0629 | 0 | Tyrosine metabolism | down |
|  | 58 | 1,2-dihydroxy-3-keto-5-methylthiopentene | C15606 | 1.9384 | 0.0596 | 0 | Cysteine and methionine metabolism | down |
|  | 59 | L-tryptophan | C00078 | 1.5359 | 0.0583 | 0 | Glycine, serine and threonine metabolism; Tryptophan metabolism; Phenylalanine, tyrosine and tryptophan biosynthesis; Aminoacyl-tRNA biosynthesis; Central carbon metabolism in cancer | down |
|  | 60 | (6r)-l-erythro-6,7-dihydrobiopterin | C00268 | 1.8902 | 0.0512 | 0 | Folate biosynthesis | down |
|  | 61 | Normetanephrine | C05589 | 1.9628 | 0.0477 | 0 | Tyrosine metabolism | down |
|  | 62 | Guanosine 5'-monophosphate | C00144 | 2.0047 | 0.0472 | 0 | Purine metabolism; cGMP-PKG signaling pathway | down |
|  | 63 | Deoxyguanosine monophosphate | C00362 | 1.9935 | 0.0468 | 0 | Purine metabolism | down |
|  | 64 | 4-methyl-5-(2-phosphonooxyethyl)thiazole | C04327 | 2.2519 | 0.0423 | 0 | Thiamine metabolism | down |
|  | 65 | S-adenosylmethionine | C00019 | 2.3759 | 0.0185 | 0 | Cysteine and methionine metabolism; Arginine and proline metabolism | down |
|  | 66 | 2-amino-1,3,4-octadecanetriol | C12144 | 2.8429 | 0.0098 | 0 | Sphingolipid metabolism | down |
| Negative Mode | 67 | 5-hydroxyindole-3-acetic acid | C05635 | 2.1011 | 98.4876 | 0 | Tryptophan metabolism | up |
|  | 68 | Phosphoenolpyruvic acid | C00074 | 2.1876 | 87.2039 | 0 | Citrate cycle (TCA cycle)；Carbon metabolism; Biosynthesis of amino acids | up |
|  | 69 | L-kynurenine | C00328 | 1.9873 | 65.4094 | 0 | Tryptophan metabolism | up |
|  | 70 | Formylkynurenine | C02700 | 2.0346 | 59.1036 | 0 | Tryptophan metabolism | up |
|  | 71 | Cysteine-glutathione disulfide | C05526 | 2.0493 | 17.7575 | 0 | Cysteine and methionine metabolism | up |
|  | 72 | (2e,6e)-farnesyl monophosphate | C20121 | 1.3062 | 14.48 | 0 | Terpenoid backbone biosynthesis | up |
|  | 73 | L-cysteine | C00097 | 1.3816 | 12.3904 | 0 | Glutathione metabolism; Pantothenate and CoA biosynthesis; Aminoacyl-tRNA biosynthesis; Carbon metabolism; Biosynthesis of amino acids | up |
|  | 74 | Α-d-glucose-1,6-bisphosphate | C01231 | 1.8112 | 11.0928 | 0 | Starch and sucrose metabolism | up |
|  | 75 | L-cysteinylglycine | C01419 | 1.5742 | 9.9 | 0 | Glutathione metabolism | up |
|  | 76 | (2r)-2,3-dihydroxypropanoic acid | C00258 | 1.4813 | 8.7777 | 0.0004 | Pentose phosphate pathway; Glycine, serine and threonine metabolism; Glycerolipid metabolism | up |
|  | 77 | 6-o-phosphono-d-gluconic acid | C00345 | 1.4826 | 8.0169 | 0.0002 | Pentose phosphate pathway; Carbon metabolism | up |
|  | 78 | 11(z)-eicosenoic acid | C16526 | 1.645 | 7.0361 | 0 | Biosynthesis of unsaturated fatty acids | up |
|  | 79 | 11(z),14(z)-eicosadienoic acid | C16525 | 1.6388 | 6.9579 | 0 | Biosynthesis of unsaturated fatty acids | up |
|  | 80 | D-ribulose 5-phosphate | C00199 | 1.1396 | 5.9596 | 0 | Pentose phosphate pathway; Riboflavin metabolism; Vitamin B6 metabolism; Biosynthesis of amino acids | up |
|  | 81 | Aminolevulinic acid | C00430 | 1.0834 | 5.6272 | 0 | Glycine, serine and threonine metabolism | up |
|  | 82 | Uridine 5'-triphosphate | C00075 | 1.3084 | 5.1833 | 0 | Pyrimidine metabolism | up |
|  | 83 | 2-c-methylerythritol 4-phosphate | C11434 | 1.4104 | 4.9677 | 0 | Terpenoid backbone biosynthesis | up |
|  | 84 | L-glutathione oxidized | C00127 | 1.4016 | 4.8929 | 0 | Glutathione metabolism | up |
|  | 85 | D-galactose | C00984 | 1.4133 | 4.4438 | 0 | Galactose metabolism; Amino sugar and nucleotide sugar metabolism | up |
|  | 86 | Gluconic acid | C00257 | 1.1587 | 3.914 | 0.0001 | Pentose phosphate pathway; Carbon metabolism | up |
|  | 87 | Myristic acid | C06424 | 1.1784 | 3.7748 | 0 | Fatty acid biosynthesis | up |
|  | 88 | 16-hydroxyhexadecanoic acid | C18218 | 1.2498 | 3.6918 | 0 | Metabolic pathways | up |
|  | 89 | D-glucose 6-phosphate | C00092 | 1.204 | 3.427 | 0 | Inositol phosphate metabolism; Central carbon metabolism in cancer | up |
|  | 90 | Erucic acid | C08316 | 1.0423 | 3.2493 | 0 | Biosynthesis of unsaturated fatty acids | up |
|  | 91 | L-glutamic acid | C00025 | 1.0222 | 2.6208 | 0 | Glutathione metabolism; Aminoacyl-tRNA biosynthesis; Carbon metabolism; Biosynthesis of amino acids; ABC transporters; FoxO signaling pathway | up |
|  | 92 | Lumichrome | C01727 | 1.0147 | 0.2995 | 0 | Riboflavin metabolism | down |
|  | 93 | Biotin | C00120 | 1.1035 | 0.2972 | 0.001 | Biotin metabolism | down |
|  | 94 | Phthalic acid | C01606 | 1.0125 | 0.2906 | 0 | ABC transporters; | down |
|  | 95 | Flavin adenine dinucleotide (fad) | C00016 | 1.0747 | 0.2857 | 0 | Riboflavin metabolism; Vitamin digestion and absorption | down |
|  | 96 | Adenosine diphosphate | C00008 | 1.1338 | 0.2827 | 0.0003 | Oxidative phosphorylation; Purine metabolism; FoxO signaling pathway; Lysosome; AMPK signaling pathway | down |
|  | 97 | 5-aminovaleric acid | C00431 | 1.0534 | 0.2719 | 0 | Lysine degradation; Arginine and proline metabolism | down |
|  | 98 | Trans-cinnamic acid | C00423 | 1.1464 | 0.2303 | 0 | Ubiquinone and other terpenoid-quinone biosynthesis; Phenylalanine metabolism | down |
|  | 99 | Citrate | C00158 | 1.2298 | 0.2245 | 0 | Citrate cycle (TCA cycle); Carbon metabolism; Biosynthesis of amino acids; Central carbon metabolism in cancer; | down |
|  | 100 | Rosmarinic acid | C01850 | 1.1104 | 0.1693 | 0 | Tyrosine metabolism | down |
|  | 101 | Alpha-ketoglutaric acid | C00026 | 1.1878 | 0.1692 | 0 | Citrate cycle (TCA cycle); Arginine biosynthesis; Alanine, aspartate and glutamate metabolism; Taurine and hypotaurine metabolism; D-Glutamine and D-glutamate metabolism; HIF-1 signaling pathway; Central carbon metabolism in cancer | down |
|  | 102 | Propanoate | C00163 | 1.5114 | 0.1603 | 0 | Nicotinate and nicotinamide metabolism | down |
|  | 103 | Inosinic acid | C00130 | 1.5767 | 0.1478 | 0 | Purine metabolism | down |
|  | 104 | Pyridoxal | C00250 | 1.5609 | 0.1363 | 0 | Vitamin B6 metabolism | down |
|  | 105 | Uridine monophosphate (ump) | C00105 | 1.6623 | 0.1324 | 0 | Pyrimidine metabolism | down |
|  | 106 | (+)-abscisic acid beta-d-glucopyranosyl ester | C15970 | 1.316 | 0.13 | 0.0033 | Metabolic pathways | down |
|  | 107 | Adenosine | C00212 | 1.4365 | 0.1199 | 0 | Purine metabolism; ABC transporters; cGMP-PKG signaling pathway; cAMP signaling pathway; Sphingolipid signaling pathway | down |
|  | 108 | Indole | C00463 | 1.4893 | 0.1156 | 0 | Tryptophan metabolism; Phenylalanine, tyrosine and tryptophan biosynthesis | down |
|  | 109 | 5-hydroxytryptophan | C00643 | 1.1151 | 0.1124 | 0 | Tryptophan metabolism | down |
|  | 110 | Tryptamine | C00398 | 1.5546 | 0.1112 | 0 | Tryptophan metabolism | down |
|  | 111 | L-tryptophan | C00078 | 1.575 | 0.108 | 0 | Glycine, serine and threonine metabolism; Tryptophan metabolism; Phenylalanine, tyrosine and tryptophan biosynthesis; Aminoacyl-tRNA biosynthesis | down |
|  | 112 | Sn-38 | C11173 | 1.4249 | 0.1061 | 0 | Drug metabolism - other enzymes | down |
|  | 113 | 4-o-(4-deoxy-beta-l-threo-hex-4-enopyranuronosyl)-beta-d-galactopyranuronic acid | C06118 | 1.5647 | 0.0946 | 0.0001 | Pentose and glucuronate interconversions | down |
|  | 114 | D-(-)-mannitol | C00392 | 1.5015 | 0.0946 | 0 | Fructose and mannose metabolism | down |
|  | 115 | Methylmalonic acid | C02170 | 1.7611 | 0.0905 | 0 | Pyrimidine metabolism; Valine, leucine and isoleucine degradation | down |
|  | 116 | Indole-3-acetic acid | C00954 | 1.434 | 0.0893 | 0 | Tryptophan metabolism | down |
|  | 117 | 20-hydroxy-(5z,8z,11z,14z)-eicosatetraenoic acid | C14748 | 1.3963 | 0.0797 | 0 | Arachidonic acid metabolism | down |
|  | 118 | Homovanillic acid | C05582 | 1.5413 | 0.0718 | 0.0001 | Tyrosine metabolism | down |
|  | 119 | S-adenosylhomocysteine | C00021 | 1.3839 | 0.0684 | 0 | Cysteine and methionine metabolism | down |
|  | 120 | Xanthosine | C01762 | 1.1708 | 0.0668 | 0 | Purine metabolism; ABC transporters | down |
|  | 121 | N-acetyl-l-phenylalanine | C03519 | 1.66 | 0.0668 | 0 | Phenylalanine metabolism | down |
|  | 122 | 2-amino-2,3,7-trideoxy-d-lyxo-hept-6-ulosonic acid | C16850 | 1.5653 | 0.065 | 0 | Phenylalanine, tyrosine and tryptophan biosynthesis | down |
|  | 123 | Phenylpyruvic acid | C00166 | 2.0417 | 0.0582 | 0 | Phenylalanine metabolism; Phenylalanine, tyrosine and tryptophan biosynthesis | down |
|  | 124 | 5'-methylthioadenosine | C00170 | 1.7025 | 0.0496 | 0 | Cysteine and methionine metabolism | down |
|  | 125 | Adenine | C00147 | 2.0855 | 0.0382 | 0 | Purine metabolism | down |
|  | 126 | Guanosine monophosphate | C00144 | 2.0963 | 0.0339 | 0 | Purine metabolism; cGMP-PKG signaling pathway | down |
|  | 127 | 3-phenyllactic acid | C05607 | 1.9035 | 0.0285 | 0 | Phenylalanine metabolism | down |
|  | 128 | Rhizocticin c | C17960 | 2.2583 | 0.0264 | 0 | Phosphonate and phosphinate metabolism | down |
|  | 129 | Deoxyguanosine monophosphate | C00362 | 2.2742 | 0.0158 | 0 | Purine metabolism | down |
